# How Do Efficient Coding Strategies Depend on Origins of Noise in Neural Circuits?

**DOI:** 10.1371/journal.pcbi.1005150

**Published:** 2016-10-14

**Authors:** Braden A. W. Brinkman, Alison I. Weber, Fred Rieke, Eric Shea-Brown

**Affiliations:** 1 Department of Applied Mathematics, University of Washington, Seattle, Washington, United States of America; 2 Department of Physiology and Biophysics, University of Washington, Seattle, Washington, United States of America; 3 Graduate Program in Neuroscience, University of Washington, Seattle, Washington, United States of America; 4 Howard Hughes Medical Institute, University of Washington, Seattle, Washington, United States of America; Duke University, UNITED STATES

## Abstract

Neural circuits reliably encode and transmit signals despite the presence of noise at multiple stages of processing. The efficient coding hypothesis, a guiding principle in computational neuroscience, suggests that a neuron or population of neurons allocates its limited range of responses as efficiently as possible to best encode inputs while mitigating the effects of noise. Previous work on this question relies on specific assumptions about where noise enters a circuit, limiting the generality of the resulting conclusions. Here we systematically investigate how noise introduced at different stages of neural processing impacts optimal coding strategies. Using simulations and a flexible analytical approach, we show how these strategies depend on the strength of each noise source, revealing under what conditions the different noise sources have competing or complementary effects. We draw two primary conclusions: (1) differences in encoding strategies between sensory systems—or even adaptational changes in encoding properties within a given system—may be produced by changes in the structure or location of neural noise, and (2) characterization of both circuit nonlinearities as well as noise are necessary to evaluate whether a circuit is performing efficiently.

## Author Summary

For decades the efficient coding hypothesis has been a guiding principle in determining how neural systems can most efficiently represent their inputs. However, conclusions about whether neural circuits are performing optimally depend on assumptions about the noise sources encountered by neural signals as they are transmitted. Here, we provide a coherent picture of how optimal encoding strategies depend on noise strength, type, location, and correlations. Our results reveal that nonlinearities that are efficient if noise enters the circuit in one location may be inefficient if noise actually enters in a different location. This offers new explanations for why different sensory circuits, or even a given circuit under different environmental conditions, might have different encoding properties.

## Introduction

Our sensory systems encode information about the external environment and transmit this information to higher brain areas with remarkable fidelity, despite a number of sources of noise that corrupt the incoming signal. Noise—variability in neural responses that masks the relevant signal—can arise from the external inputs to the nervous system (e.g., in stochastic arrival of photons at the retina, which follow Poisson statistics) and from properties intrinsic to the nervous system, such as variability in channel gating, vesicle release, and neurotransmitter diffusion (reviewed in [1]). This noise places fundamental limits on the accuracy with which information can be encoded by a cell or population [2–5]. An equally important consideration, however, is that noise dictates which processing strategies adopted by the nervous system will be most effective in transmitting signal relative to noise.

Efficient coding theory has been an important principle in the study of neuroscience for over half a century, and a number of studies have found that neural circuits can encode and transmit as much useful information as possible given physical and physiological constraints [6–13]. Foundational work by Laughlin successfully predicted the function by which an interneuron in the blowfly eye transformed its inputs [7]. This and other early work prompted a myriad of studies that considered how neurons could make the most efficient use of their output range in a variety of systems and stimulus conditions [14–19]. Efficient coding theory has played an important role in how we interpret biological systems. However, one cannot know how efficiently a neuron or population is encoding its inputs without understanding the sources of noise present in the system. Several previous studies have recognized noise as an important factor in determining optimal computations [8, 11, 12, 20, 21]. These and related studies of efficient coding often make strong assumptions about the location of noise in the system in question, and these assumptions are typically not based on direct measurements of the underlying noise sources. For example, noise is often assumed to arise at the output stage and follow Poisson statistics. Yet experimental evidence has shown that spike generation itself is near-deterministic, implying that most noise observed in a neuron’s responses is inherited from earlier processing stages [22–24]. Indeed, several different sources of noise may contribute to response variability, and the relative contributions of these noise sources can change under different environmental and stimulus conditions [25–27]. Importantly, the results of efficient coding analyses depend on the assumptions made about the locations of noise in the system in question, but there has been to date no systematic study of the implications that different noise sources have for efficient coding strategies. In particular, identifying failures of efficient coding theory—i.e., neural computations that do not optimally transform inputs—necessitates a broad understanding of how different sources of noise alter efficient coding predictions.

Here, we consider how the optimal encoding strategies of neurons depend on the location of noise in a neural circuit. We focus on the coding strategies of single neurons or pairs of neurons in feedforward circuits as simple cases with physiologically relevant applications. Indeed, early sensory systems often encode stimuli in a small number of parallel channels, including in vision [28–30], audition [31], chemosensation [32], thermosensation [33], and somatosensation [34]. We build a model that incorporates several different sources of noise, relaxing many of the assumptions of previously studied models, including the shape of the function by which a neuron transforms its inputs to outputs. We determine the varied, and often competing, effects that different noise sources have on efficient coding strategies and how these strategies depend on the location, magnitude, and correlations of noise across neurons. Much of the efficient coding literature is impacted by these results. For example, Laughlin’s predictions assume that downstream noise is identical for all responses; when this is not true, a different processing strategy will be optimal. Other recent work, considering such questions as when it is advantageous to have diverse encoding properties in a population and when sparse firing is beneficial, bears reinterpretation in light of these results [21, 35]. Our work demonstrates that understanding the sources of noise in a neural circuit is critical to interpreting circuit function.

## Results

Our goal is to understand how diverse noise sources shape a neural circuit’s optimal encoding strategies. We determine the optimal nonlinearities using two complementary approaches. First, we take variational derivatives of the mean squared error (MSE) between the true input and a linear estimate of the input to derive a system of equations for the exact optimal nonlinearities. We constrain the output of the nonlinearities to fall within a fixed range to reflect the limited dynamic range of neurons, but aside from this, we make no assumptions about the shape of the nonlinearities. Second, we simulate the model by parametrizing the nonlinearities and numerically determining the parameter values of the nonlinearity that best encode the stimulus. With this approach, we can use more complex measures of coding fidelity, such as mutual information (MI), as our criterion for optimality (see Methods for details).

We first describe a feedforward neural circuit model that incorporates three potential sources of noise. We then describe how a single pathway should allocate its response range to optimally encode its inputs, showing that optimal strategies depend strongly on where noise enters the circuit. Finally, we extend our model to include two parallel pathways, reflecting a common architecture in sensory systems. We consider how dual pathways should parcellate the range of inputs, namely the factors that determine to what extent they should encode overlapping regions of the input distribution and whether they should have the same or different response polarities.

### Circuit model

**Fig 1:**
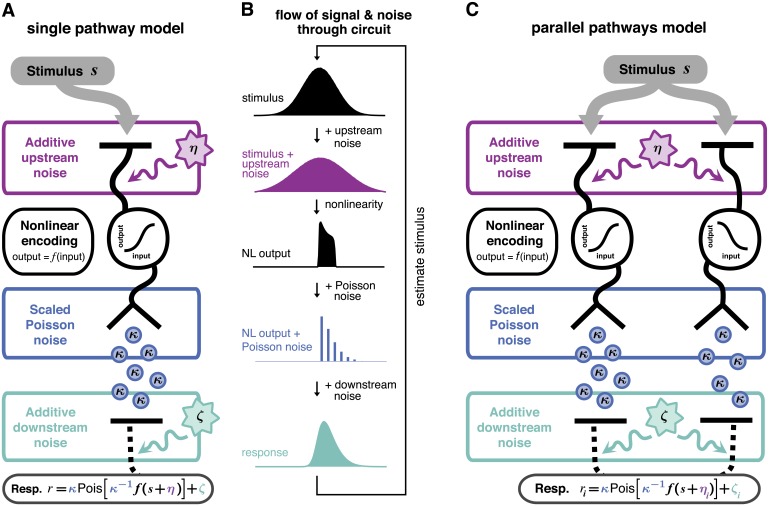
Model of neural encoding with three sources of noise. **A:** Model schematic for a single pathway. An input *s* is directly corrupted by some noise *η*, and then transformed nonlinearly by *f*(⋅). The nonlinear processing stage sets the mean of a scaled Poisson response with variance equal to *κ* times the mean response. This response is corrupted by additional additive downstream noise *ζ* to give a total response *r*. **B:** Transformed stimulus distribution at each stage of the model. **C:** Model schematic for two parallel pathways. Noise upstream and downstream of the nonlinearity may be correlated across neurons. For schematic purposes, we have drawn all signal processing steps as though they are contained within a single neuron, but each pathway could more generally represent signal processing spread out across multiple neurons.

The model is schematized in Fig 1, and is detailed below. We constructed this model with retinal circuitry in mind, though the model could be reinterpreted to represent other primarily feedforward early sensory systems, or even small segments of cortical circuitry. We begin with a simple feature of neural circuits that captures a ubiquitous encoding transformation: a nonlinear conversion of inputs to outputs. Nonlinear processing arises from several biological processes, such as dendritic integration, vesicle release at the synapse, and spike generation [36, 37]. Such nonlinearities appear in most neural coding models (such as the commonly used linear-nonlinear-poisson (LNP) models or generalized linear models [38–40]). Although there are likely several sites with some level of nonlinear processing in the retinal circuitry, there is a single dominant nonlinearity at most light levels which can be localized to the output synapse of the bipolar cells [41]. Our goal is to determine the shape of the nonlinearity in this model that most faithfully encodes a distribution of inputs—i.e., the optimal encoding strategy. Indeed, in the retina, the shape of this nonlinearity has been shown to adapt under different stimulus conditions, suggesting that this adaptation might serve to improve encoding of visual stimuli as environmental conditions (and hence noise) change [18, 42].

The pathway receives an input signal or stimulus *s*, which is drawn from the standard normal distribution. Generally, an individual value of *s* can represent any deviation from the mean stimulus value, and the full distribution of *s* represents the set of inputs that might be encountered over some time window in which the circuit is able to adapt. In the context of the retinal circuitry, *s* can be understood as the contrast of a small region, or pixel, of the visual stimulus. The contrast in this pixel might be positive or negative relative to the ambient illumination level. The full distribution of *s* would then represent the distribution of contrasts encountered by this bipolar cell as the eye explores a particular scene. (We use Gaussian distributions here for simplicity in analytical computations, though similar results are obtained in simulations with skewed stimulus distributions, similar to the distributions of pixel contrast of natural scenes [43].) We assume the distribution of *s* is fixed in time. If properties of the signal distribution varied randomly in time (for example, if the variance of possible signals the circuit receives fluctuates between integration times), over long times the circuit would see an effectively broader distribution due to this extra variability. Conversely, if the particular visual scene being viewed or other environmental conditions change suddenly, the input distribution as a whole (for example, the range of contrasts, corresponding to the width of the input distribution) also changes suddenly. Therefore we expect the shape of the optimal nonlinearity to adapt to this new set of signal and noise distributions. We do not model the adaptation process itself; our results for the optimal nonlinearity correspond to the end result of the adaptation process in this interpretation.

We incorporate three independent sources of noise, located before, during, and after the nonlinear processing stage (Fig 1A and 1B). The input stimulus is first corrupted by upstream noise *η*. This noise source represents various forms of sensory noise that corrupt signals entering the circuit. This might include noise in the incoming stimulus itself or noise in photoreceptors. The strength of this noise source is governed by its variance, $\sigma_{\text{up}}^{2}$. The signal plus noise (Fig 1B, purple) is then passed through a nonlinearity *f*(⋅), which sets the mean of a scaled Poisson process with a quantal size *κ*. The magnitude of *κ* determines the contribution of this noise source, with large values of *κ* corresponding to high noise. This noise source captures quantal variations in response, such as synaptic vesicle release, which can be a significant source of noise at the bipolar cell to ganglion cell synapse [26]. Finally, the scaled Poisson response is corrupted by downstream noise *ζ* (with variance $\sigma_{\text{down}}^{2}$) to obtain the output response (Fig 1B, green). This source of noise captures any variability introduced after the nonlinearity, such as noise in a postsynaptic target. In the retina, this downstream noise captures noise intrinsic to a retinal ganglion cell, and the final output of the model is the current recorded in a ganglion cell. If the sources of upstream and downstream noise are independent (e.g., photoreceptor noise and retinal ganglion cell channel noise, respectively), then the two kinds of noise will be uncorrelated in a feedforward circuit like we model here. Lateral input from other channels, which we do not consider, could potentially introduce dependence between upstream and downstream noise. Feedback connections operating on timescales within a single-integration window could also potentially introduce correlations between additive upstream and downstream noises. However, while such connections could be important in cortical circuits, they are not significant in the sensory circuits that inspired this model, so we assume independent upstream and downstream noise in this work. For further biological interpretation of the model, see Discussion.

We begin by studying a model of a single pathway. We then consider how two pathways operating in parallel ought to divide the stimulus space to most efficiently code inputs. These models are constructed of two parallel pathways of the single pathway motif (Fig 1C), with the addition that noise may be correlated across both pathways. The study of two parallel channels is motivated by the fact that a particular area of visual space is typically encoded by paired ON and OFF channels with otherwise similar functional properties, but similar parallel processing occurs throughout early sensory systems and in some cortical areas [29, 31, 32]. We will return to further discussion of parallel pathways in the second half of the Results.

### Optimal coding strategies for single pathways

We begin with the case of a single pathway. For simplicity, we start with cases in which one of the three noise sources dominates over the others. Considering cases in which a single noise source dominates isolates the distinct effects of each noise source on the optimal nonlinearity. We then show that these same effects govern how the three noise sources compete in setting the optimal nonlinearity when they are all of comparable magnitude.

#### Upstream noise decreases slope of optimal nonlinearity to encode broader range of inputs

**Fig 2:**
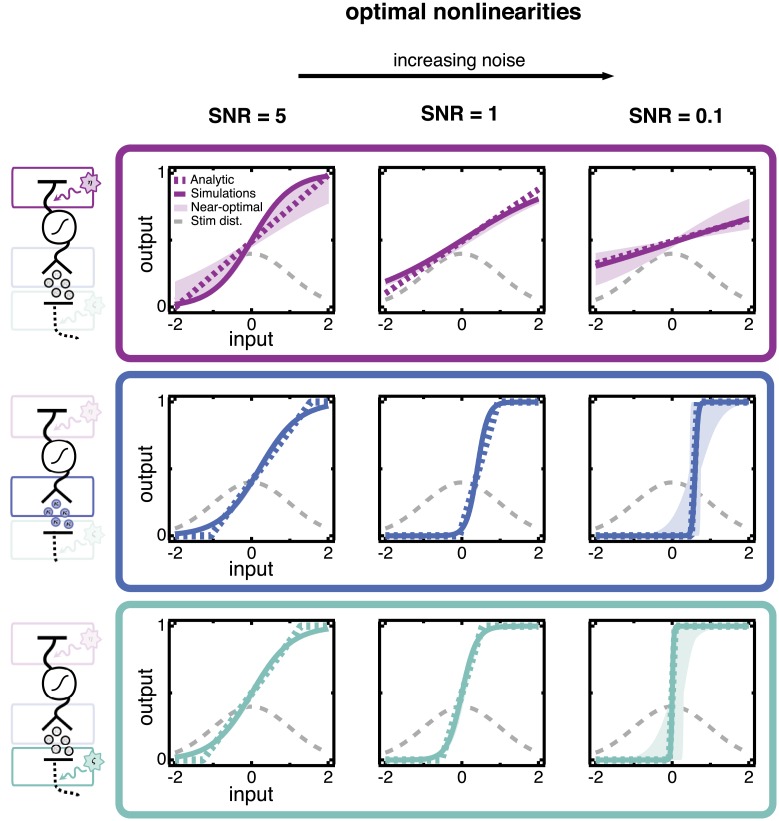
Optimal nonlinearities when one noise source dominates, found by minimizing the mean squared error (MSE) of a linear estimator. Each row shows three separate cases in which a single source of noise dominates. The dominant noise source is indicated by the highlighted source in the circuit schematics left of each row. The overall level of noise is quantified by the signal-to-noise ratio (SNR), which is fixed in each column. The SNR is largest in the leftmost column and smallest in the rightmost column; i.e., the strength of the *noise* increases toward the right. The shape of the optimal nonlinearity changes markedly depending on which noise source dominates the circuit, *even when the overall signal-to-noise ratio of model responses is the same*. Analytical results (dashed colored lines) and simulations with sigmoidal nonlinearities (solid lines) are shown. The stimulus distribution (dashed gray curve) is also shown for reference. Shaded regions encompass nonlinearities that perform within 1% of the minimum mean squared error of the optimal sigmoidal nonlinearity. The SNR is computed as the variance of the signal (the variance, across all inputs, of the average response to a given input) divided by the variance of the noise (the average variance in responses to a given input); see Methods.

In Fig 2, we plot the optimal nonlinearities for cases in which one of the noise sources dominates the others. For each noise source, we show results for small, intermediate, and large values of the signal-to-noise ratio (SNR) of model responses. Importantly, the SNR is matched within columns of Fig 2, allowing for a direct comparison of the effects of different noise sources. We present both analytical results (dashed lines) for optimal nonlinearities constrained only by the assumption of fixed dynamic range, and results using parametrized nonlinearities of a sigmoidal form (solid lines). We show only optimal “ON” nonlinearities (nonlinearities that increase response strength as stimulus strength increases) in this section for simplicity; the mirror-image “OFF” nonlinearities (which decrease response strength as stimulus strength increases) are mathematically equivalent and result in identical values of MSE or MI.

We begin with the case in which the upstream noise dominates (Fig 2, top row). The optimal nonlinearities are centered around the most likely stimulus and have progressively lower slopes for greater upstream noise variance. Upstream noise is added directly to the stimulus and hence cannot be removed by any nonlinear transformation. The optimal strategy in this case is to ensure that the limited range of outputs is used to encode the entire range of inputs. Increasing upstream noise effectively broadens the input distribution, and decreasing the slope of the nonlinearity compensates for this broadening. Quantitatively, we find that the effect of upstream noise is captured entirely by normalizing the inputs (stimulus plus upstream noise) by their standard deviation $\left(\sqrt{\sigma_{s}^{2}+\sigma_{\text{up}}^{2}}\right)$ (see Methods). In other words, nonlinearities are simply scaled versions of each other that overlay entirely when normalized by the effective range of inputs (stimulus plus noise) they receive.

**Fig 3:**
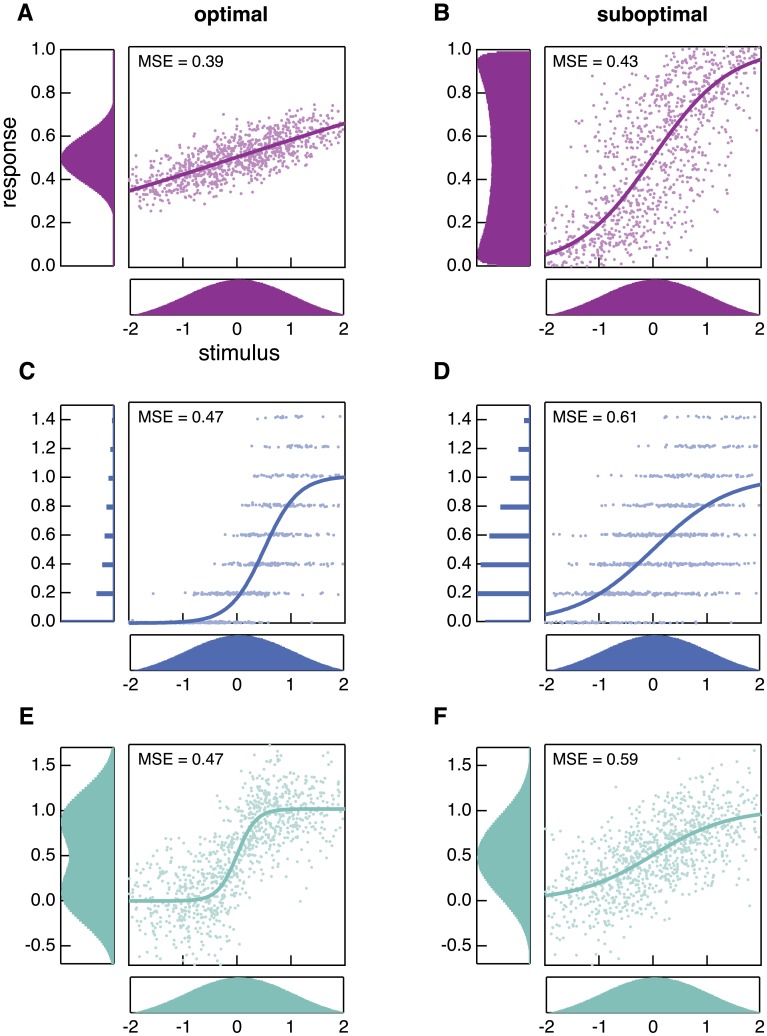
Responses produced by optimal (left column) and suboptimal (right column) nonlinearities. Each row shows a different set of noise conditions in which a single source of noise is dominant (i.e., upstream noise dominates in panels **A** and **B**, Poisson noise in **C** and **D**, and downstream noise in **E** and **F**). Markers show 1,000 points randomly selected from the stimulus distribution (bottom subpanels) and the corresponding responses that are produced by the nonlinearity (solid line). Different nonlinearities produce very different response distributions (left subpanels). These particular suboptimal nonlinearities are chosen for illustrative purposes, to highlight qualitative features of the optimal nonlinearities.

It is instructive to see the responses produced by both optimal and suboptimal nonlinearities to clarify this intuition (Fig 3). A suboptimal nonlinearity (Fig 3B) has a relatively steep slope, which results in a large number of inputs producing either maximal or minimal responses. As a result, the response distribution shows peaks near the edges of the response range. The optimal nonlinearity (Fig 3A) has a shallower slope which prevents saturation of the outputs.

#### Poisson noise shifts the optimal nonlinearity so that low-noise responses encode most likely stimuli

We next isolate the effect of the scaled Poisson noise, by considering the case where its magnitude dominates the other noise sources (Fig 2, middle row). Increasing *κ* increases the slope of the optimal nonlinearity and shifts it off-center. The scaled Poisson noise has variance proportional to the mean response. Thus, stimuli that elicit the weakest responses also generate the lowest noise. The offset of the optimal nonlinearity associates the least noisy range of outputs, near the base of the nonlinearity, with the most probable stimuli.

A suboptimal nonlinearity (Fig 3D) maps a significant proportion of inputs to medium and high responses, which are noisy. Conversely, the optimal nonlinearity (Fig 3C) maps a large proportion of inputs to lower response values, including many to 0, which has no associated Poisson noise. This comes at the cost of compressing many stimuli to the same response value, but in terms of decoding error is more than compensated for by decreased levels of noise.

We chose to model this source of noise as following Poisson statistics, as several lines of evidence suggest that vesicle release at synapses in the retina is well-described as Poissonian [44, 45]. However, we also tested to what extent the results here depend on this particular assumption. We investigated how optimal nonlinearities change for two additional types of noise that might be associated with the nonlinear stage: (1) multiplicative Gaussian noise, where the variance is proportional to the output of the nonlinearity, and (2) vesicle release that follows a binomial distribution, where the output of the nonlinearity determines the probability of release. In both cases (and for both criteria for optimality, MSE and MI), results are qualitatively similar to those presented here (see S1 Fig). The trends were necessarily identical for the Poisson and multiplicative Gaussian noises, which both have variances proportional to the nonlinearity. For a linear stimulus estimator, as used in this work, the MSE depends only on the mean and covariances of the nonlinear-stage noise—higher order statistics do not affect the shape of the optimal nonlinearity determined by minimizing the MSE. Hence, any circuit in which the mean and variances of the response are proportional to the nonlinearity will yield the same optimal nonlinearities.

#### Downstream noise steepens slopes to improve discriminability of responses

Finally, we study the case where the downstream noise dominates other noise sources (Fig 2, bottom row). Here, the optimal nonlinearity remains centered for a range of noise strengths but becomes markedly steeper as the variance of downstream noise increases. Steepening the slope amplifies changes in the response with respect to the stimulus, while leaving the downstream noise unchanged. The result is a greater signal-to-noise ratio for those stimuli that fall near the midpoint of the nonlinearity. Placing the nonlinearity in the center of the stimulus distribution ensures that the most likely stimuli will be the most discriminable. This differs from the case of upstream noise, where the slope becomes less steep as noise increases. Unlike upstream noise, which corrupts the stimulus directly, the signal and downstream noise can be differentially amplified to improve the SNR.

The optimal nonlinearity for large downstream noise (Fig 3E) has a steep slope. Responses corresponding to stimuli above versus below the mean can be clearly distinguished from the response distribution. This improved discriminability comes at the cost of encoding a smaller range of inputs, but this is compensated for by the improved discriminability for inputs that fall within the range encoded by the nonlinearity. A suboptimal nonlinearity (Fig 3F), on the other hand, results in relatively poor discrimination of a broader range of inputs: small differences in inputs lead to small differences in outputs, which are overcome by the downstream noise. If the downstream noise is much larger than the typical response sizes, the nonlinearity essentially becomes an all-or-nothing response; i.e., the downstream noise is so large that the response can only provide information about whether or not the stimulus is greater or less than the mean. Some of this effect can be seen in the example shown in Fig 3E, with the response distribution becoming bimodal.

To ensure that our results are not dependent on our criterion for optimality (i.e., minimizing the mean squared error of a linear estimator), we also used simulations to find the optimal nonlinearities that maximize the mutual information between stimulus and response (see Methods for details). Nonlinearities that maximize the MI show the same trends as those that minimize MSE (S2 Fig; compare to Fig 2).

#### Noise sources compete to shape the nonlinearity

The results in Fig 2 demonstrate that optimal nonlinearities have very different shapes when different noise sources dominate, even if they have the same overall “strength” (i.e., induce the same SNR). In particular, upstream and downstream noise have opposite effects on the optimal nonlinearity, even though they are both Gaussian and additive at their source.

**Fig 4:**
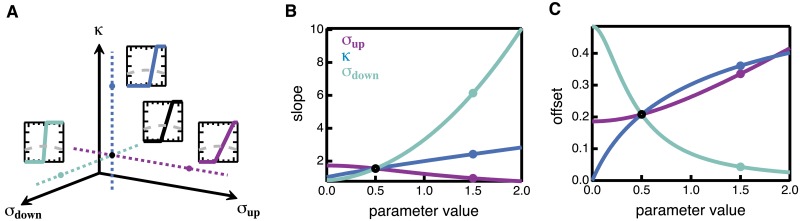
Optimal nonlinearities for cuts through parameter space. **A:** Schematic showing regions of 3-dimensional parameter space: *σ*_up_ (purple), *σ*_down_ (green), and *κ* (blue). Insets show the optimal nonlinearity corresponding to the like-colored point, along with the stimulus distribution (dashed gray curves). **B:** Slope of the optimal nonlinearity as each parameter is varied along the corresponding dashed axis in **A**. **C:** Offsets plotted in the same manner as **B**. Slopes and offsets are obtained from the exact solutions of the model.

We now show that the same trends occur when there are multiple noise sources of comparable strengths. In this case the optimal nonlinearities lie between the solutions shown above and change smoothly as the noise parameters are varied. Fig 4 takes three cuts through the (*σ*_up_, *κ*, *σ*_down_) parameter space and shows how the optimal nonlinearity changes as one moves in a particular direction. Once again, we see that different noise sources often have opposite effects on features of the optimal nonlinearity. This highlights the importance of considering model assumptions about where noise enters in a circuit. When trying to determine whether a given circuit is operating optimally, one can arrive at opposite conclusions depending on where noise is assumed to be present. This is an important point given that there are many different ways in which noise is commonly incorporated into neural models (e.g., Poisson noise in the nonlinearity or additive Gaussian noise) and these assumptions are not frequently based on knowledge of noise location in the corresponding biological circuit.

### Optimal coding strategies for parallel pathways

Information in many sensory systems is encoded in parallel pathways. In vision, for example, inputs are encoded by both ON cells and OFF cells. In audition, an incoming stimulus is encoded in many parallel channels, each encoding a particular frequency band. Allowing for multiple parallel channels raises fundamental questions about how these resources should be allocated: should multiple channels have the same or different response polarities? Should an input be encoded in multiple channels redundantly, or should different channels specialize in encoding a particular range of inputs? To understand these tradeoffs, we solved our model for the optimal nonlinearities for a pair of parallel pathways, the simplest case in which these questions can be investigated. Indeed, in many cases, a small number of sensory neurons are responsible for carrying the relevant signal [46–49].

Our circuit model for multiple pathways comprises parallel copies of the single pathway model (Fig 1C), with the additional detail that both upstream and downstream noise may be correlated across pathways. We show below that the sign and strength of these correlations can strongly affect optimal encoding strategies. To focus on the effects of noise on optimal encoding strategies, we added complexity to the noise structure, while making significant simplifications in the stimulus structure. In particular, we assume that both channels receive the same stimulus. Correlated but non-identical stimuli in the two channels would likely affect optimal encoding strategies, but we did not explore this possibility and leave it as a direction for future inquiry.

We discuss the parallel pathway results in the following order: first, we discuss the possible pairs of nonlinearities, which are richer than the single-pathway case. We then discuss the functional effects that each of the parameters, or in some cases combinations of parameters, has on the shapes of the nonlinearities, with a focus on which parameter regimes favor highly overlapping versus minimally overlapping encoding of inputs (hereafter referred to as “overlapping” and “non-overlapping”). Finally, we discuss factors that determine whether a circuit should encode inputs with channels of opposite polarity versus channels of the same polarity.

#### Multiple optimal coding strategies exist—“ON-OFF” and “ON-ON” pairs

Similar to the single pathway case above, solving the model reveals that each nonlinearity may be of the ON or OFF type, allowing for four combinations of pairs: ON-ON, ON-OFF, OFF-ON, and OFF-OFF. Note that we did not make assumptions about the shape of the optimal nonlinearities, other than restricting the output range. In particular, we did not explicitly impose monotonicity of the nonlinearities, though this may be a consequence of our choice of linear stimulus estimator. Non-monotonic nonlinearities have been proposed to serve a variety of computational functions [50–56], and understanding under what conditions they are optimal is an interesting and important direction for future studies; here we focus on the monotonic nonlinearities obtained from our analytic approach.

**Fig 5:**
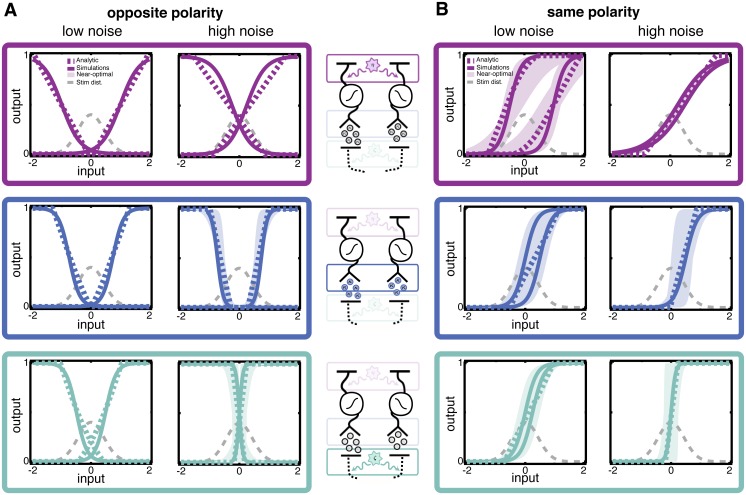
Optimal nonlinearities for a circuit with two parallel pathways, found by minimizing mean squared error (MSE) of a linear estimator. **A:** Optimal nonlinearities for circuits in which pathways are of opposite polarity. Each row corresponds to a case in which one particular noise source is dominant. The dominant noise source is indicated by the highlighted source in the schematics at the center of the figure. As in Fig 2, solid curves are the optimal sigmoidal nonlinearities and colored dashed curves are optimal nonlinearities obtained analytically. The gray dashed curves represent the stimulus distribution. Shaded regions represent the range of sigmoidal nonlinearities that perform within 1% of the mean squared error of the optimal sigmoidal nonlinearities. **B:** Same as **A** but for pathways of the same polarity. We address the relative coding efficiency of the different polarities in the section “Globally optimal strategies.”

The ON-ON and OFF-OFF pairs are related by symmetry of the model and are equivalent, as are the ON-OFF and OFF-ON pairs. However, the ON-OFF and ON-ON pairs are *not* equivalent and have different decoding error (MSE) (similar to Ref. [56]). All ON-OFF pairs are anti-symmetric (*f*_1_(*z*) = *f*_2_(− *z*)), with varying degrees of overlap between nonlinearities (Fig 5A). However, ON-ON nonlinearities split into separate subclasses: identical ON-ON pairs are two copies of the same nonlinearity, *f*_1_(*z*) = *f*_2_(*z*) and hence overlap entirely, while non-identical ON-ON pairs split their thresholds and overlap only in the tails, similar to the configurations considered in several recent studies [21, 56]. For some noise conditions, identical and non-identical ON-ON pairs are co-existing analytical solutions of the model; the MSE of the two solutions need not be equal. The non-identical subclass only exists when the circuit can lower the MSE by splitting the thresholds. The situation is simpler for ON-OFF pairs, for which we only observe a single solution for each noise condition.

We first present results for both ON-OFF and ON-ON classes of solutions, showing how different noise sources shape optimal nonlinearities in a circuit with two parallel channels. The qualitative effects of each noise source are consistent across ON-OFF and ON-ON architectures. We then show which parameter determines whether same polarity or opposite polarity solutions are globally optimal.

#### Highly correlated inputs favor non-overlapping encoding, uncorrelated inputs favor overlapping encoding

To fully understand how the nonlinearity pairs change when a given noise source dominates responses, we need to be precise about what we mean when we say upstream noise or downstream noise dominates. The overall impacts of the noise sources are not simply the variances $\sigma_{\text{up}}^{2}$ and $\sigma_{\text{down}}^{2}$ but are also influenced by the degree of correlation between the noise sources across pathways, *ρ*_up_ and *ρ*_down_. As we will make clear, these correlations dictate the effective noise level, i.e., the extent to which noise interferes with signal decoding (see [2] for relevant discussion of noise correlations).

We find that upstream noise parameters (both variance and correlations) determine the degree to which optimal nonlinearities overlap, encoding some inputs in both channels. This is true regardless of whether nonlinearities in the two pathways are of the same polarity (“ON-ON”) or opposite polarities (“ON-OFF”). We can gain insight into this result by first considering the effects of the two upstream noise terms ($\sigma_{\text{up}}^{2}$ and *ρ*_up_) individually. First, consider the effects of changing the magnitude of upstream noise if it is uncorrelated across channels. If the magnitude of noise is small (small $\sigma_{\text{up}}^{2}$), one channel can reliably encode a stimulus, and the most favorable strategy is for each pathway to encode a different range of inputs. This allows the largest range of inputs to be encoded. If $\sigma_{\text{up}}^{2}$ is large, however, it is beneficial to redundantly encode the input in both channels, as averaging can be used to better recover the stimulus, given that the noise is not (positively) correlated.

Now consider the effects of varying amounts of noise correlations, *ρ*_up_, for a fixed noise magnitude. Weak upstream noise correlations (*ρ*_up_ ≈ 0) mean that averaging can be beneficial, as the noise is independent in each channel. This favors overlapping encoding. Strong noise correlations (*ρ*_up_ ≲ 1), on the other hand, result in coherent shifts of the stimulus in both pathways, making it difficult to distinguish what part of the input is signal and what part of the input is noise. Encoding highly correlated inputs (including highly correlated noise) with two overlapping nonlinearities offers no advantage over encoding with a single nonlinearity. The better strategy, then, is to use each pathway to encode a different range of inputs, maximizing coverage of the input space.

It turns out that these changes can be wholly captured by the correlation coefficient of the *total inputs* (stimulus plus noise) to each channel,

$$\begin{array}{c} \rho_{\text{eff}}\equiv \frac{\left\langle \left(s+\eta_{1}\right)\left(s+\eta_{2}\right)\right\rangle }{\sqrt{\left\langle {\left(s+\eta_{1}\right)}^{2}\right\rangle \left\langle {\left(s+\eta_{2}\right)}^{2}\right\rangle }}=\frac{\sigma_{s}^{2}+\sigma_{\text{up}}^{2}\rho_{\text{up}}}{\sigma_{s}^{2}+\sigma_{\text{up}}^{2}}. \end{array}$$ (1)

i.e., $\sigma_{\text{up}}^{2}$ and *ρ*_up_ do not independently control the degree of overlap of the nonlinearities. Analytical calculations (see Methods) show that when the input to the nonlinearities is rescaled by its standard deviation ($\sqrt{\sigma_{s}^{2}+\sigma_{\text{up}}^{2}}$, as in the single cell case), the dependence on *σ*_s_, *σ*_up_, and *ρ*_up_ enters only through the effective parameter combination *ρ*_eff_.

In summary, when inputs (stimulus plus noise) to parallel channels are uncorrelated, having each channel cover overlapping regions of the input space provides two distinct estimates of the stimulus. These estimates are combined to produce a better overall estimate, so encoding the stimulus in both channels is favorable. When inputs are highly correlated or when noise is low, the circuit cannot achieve better performance by encoding the input in two overlapping channels than it can with a single channel, so it is best to have each nonlinearity cover a different range of inputs to encode as large a range of stimuli as possible.

#### Poisson variability biases both channels to encode most frequent stimuli

Increasing the Poisson strength *κ* has opposite effects for ON-OFF versus ON-ON pairs (Fig 5, center row) in terms of the degree of overlap in encoding. For ON-OFF pairs, increasing *κ* pushes the nonlinearities apart, while for ON-ON pairs increasing *κ* pulls nonlinearities back together. However, both of these effects are manifestations of the principle that the most reliable responses should encode the most likely stimuli, just as in the single-pathway case. Because the variance of responses due to Poisson variability is lowest near the base of the nonlinearity, increasing *κ* biases the base of the nonlinearities to be positioned near the peak of the stimulus distribution. For ON-OFF nonlinearities, this effectively pushes the nonlinearities apart, while ON-ON nonlinearities are drawn back together. In both ON-OFF and ON-ON pairs, the slope of the nonlinearities increases to improve discriminability of inputs, as in the single-pathway case.

#### Downstream noise variance steepens nonlinearities

High downstream noise steepens the nonlinearities and drives the center of the nonlinearity back towards the most likely inputs (Fig 5, bottom row). However, even for large noise, nonlinearities may have varying degrees of overlap, depending on the values of *κ* or *ρ*_eff_. As such, the downstream noise does not have a significant influence on the degree of overlap of the encoding strategies.

As in the case of a single pathway, results obtained by maximizing the mutual information are qualitatively similar to those obtained by minimizing the mean squared error of a linear estimator (S3 Fig; compare to Fig 5).

#### Competition between noise sources

**Fig 6:**
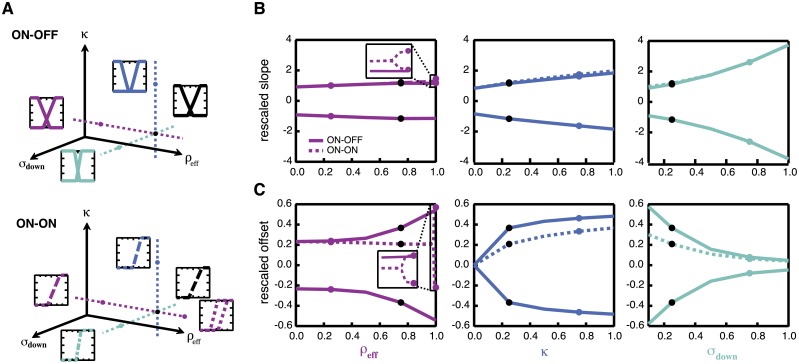
Optimal ON-OFF or ON-ON nonlinearities for slices through parameter space. **A:** Schematics showing regions of the effective 3-dimensional parameter space: total input correlation *ρ*_eff_ (purple), scaled Poisson strength *κ* (blue), and downstream noise standard deviation *σ*_down_ (green). Insets show the optimal nonlinearity corresponding to the like-colored point. Top schematic: optimal ON-OFF nonlinearities, bottom: optimal ON-ON nonlinearities. **B:** Slopes of the optimal nonlinearities (rescaled by $\sqrt{\sigma_{s}^{2}+\sigma_{\text{up}}^{2}}$) for ON-OFF (solid) and ON-ON (dashed) solutions as each parameter is varied along the corresponding dashed axis in **A**. **C:** Offsets (rescaled by $1/\sqrt{\sigma_{s}^{2}+\sigma_{\text{up}}^{2}}$) plotted in the same manner as **B**. Insets in the *ρ*_eff_ plots are zoomed in to resolve the splitting of the identical ON-ON solution into the non-identical ON-ON solution. Rescaled slopes and offsets are obtained from numerical solutions of the analytic model; see Methods for details about rescaling.

To study the competition between noise sources when there is not a clear dominant source, we sweep along cuts in the (*ρ*_eff_, *κ*, *σ*_down_) parameter space, similar to Fig 4. Here, however, we focus on how the *rescaled* slopes and offsets change under different noise conditions. As described above, after rescaling by the standard deviation of the input ($\sqrt{\sigma_{s}^{2}+\sigma_{\text{up}}^{2}}$), the effects of the three stimulus and upstream noise parameters can be combined into a single parameter *ρ*_eff_ that determines the shape of the optimal nonlinearity. We focus on the rescaled slopes (slope multiplied by $\sqrt{\sigma_{s}^{2}+\sigma_{\text{up}}^{2}}$) and rescaled offsets (offset divided by $\sqrt{\sigma_{s}^{2}+\sigma_{\text{up}}^{2}}$) in order to present the effects of the single parameter *ρ*_eff_. The Poisson strength *κ* and the downstream noise standard deviation *σ*_down_ have similar effects on the paired nonlinearities as they do in the case of a single pathway. Poisson noise increases the slope (Fig 6B, middle subpanel) and shifts the nonlinearities off-center (Fig 6C, middle subpanel). Downstream noise also steepens the slope (Fig 6B, right subpanel) and centers the nonlinearities (Fig 6C, right subpanel). The total input correlation *ρ*_eff_ has relatively little effect on the rescaled slope (Fig 6B, left subpanel), but significantly impacts the offset (Fig 6C, left subpanel). The separation of the offsets of the ON-OFF nonlinearities (solid lines) increases as *ρ*_eff_ increases towards 1, reflecting an increase in the degree of non-overlapping encoding. Under most noise conditions, the two ON-ON nonlinearities (dashed lines) are identical and hence encode inputs redundantly; however, as *ρ*_eff_ approaches 1, the offsets split, switching from the identical ON-ON subclass to the non-identical ON-ON subclass. For nonlinearities of the same polarity, this splitting is in competition with both the scaled Poisson noise and the downstream noise, which bias the base of the nonlinearities back towards the center of the stimulus distribution (compare dashed lines in subpanels across Fig 6). If *ρ*_eff_ is not large enough, as is the case for the cuts along the *κ* and *σ*_down_ dimensions in Fig 6, no splitting occurs. Splitting of ON-ON nonlinearities only occurs for a narrow range of *ρ*_eff_ between 0.9 − 1.0; this range shrinks as the strength of the Poisson or downstream noise grow. For zero Poisson and downstream noise, the range is consistent with the model of [21].

These plots demonstrate that, as in the single pathway case, the different noise sources are often in direct conflict with each other and result in qualitatively different nonlinearities. Furthermore, the effects of upstream noise correlations differ from those of downstream noise correlations, further described below.

#### Globally optimal strategies: Downstream noise determines polarity of nonlinearities

So far, we have investigated how different noise regimes shape the ON-OFF and ON-ON nonlinearities, without regard to which strategy is the globally optimal solution. We find that downstream noise correlations primarily determine the relative efficiency of ON-OFF nonlinearities compared to ON-ON nonlinearities.

**Fig 7:**
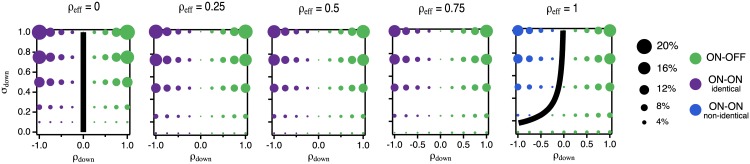
Dependence of solution polarity on noise parameters. Each panel shows the optimal solution type (indicated by color) as a function of *σ*_down_ and *ρ*_down_ for a particular value of *ρ*_eff_. Dot size indicates the percent decrease in MSE between the globally optimal solution and the best solution of a different type (ON-ON where ON-OFF is optimal, and ON-OFF where ON-ON is optimal). Dots show results from numerical solution of integral equations for the exact nonlinearities; black lines show analytic predictions for boundaries at which ON-ON and ON-OFF solutions are equally optimal. The parameter *κ* has relatively little influence on the qualitative features of this plot, so for simplicity, we only show cases where *κ* = 1.0. The crossover from identical to non-identical ON-ON solutions occurs between *ρ*_eff_ ≈ 0.9 − 1.0; strong downstream noise or Poisson strength reduces the range over which splitting occurs. The rescaled nonlinearities and globally optimal strategy depend only on the parameters *ρ*_eff_, *κ*, *σ*_down_ and *ρ*_down_; the dependence on *σ*_s_, *σ*_up_, and *ρ*_up_ enters only through *ρ*_eff_. See Methods for details.

Fig 7 maps out the optimal strategy in each region of parameter space for an exemplary value of *κ*, with different values of *ρ*_eff_ for each panel. Green points indicate that the ON-OFF strategy is optimal, while purple or blue points indicate that identical ON-ON or non-identical ON-ON strategies, respectively, are optimal. The size of the dots indicates the percent difference in MSE between the globally optimal solution and the best solution of a different type (e.g., if ON-OFF is optimal, percent difference between the optimal ON-OFF solution, and the best possible ON-ON solution).

We see that downstream noise correlations impact the relative mean squared error of ON-OFF versus ON-ON encoding strategies: ON-OFF pairs are generally optimal when the downstream noise is positively correlated across pathways, while ON-ON pairs are generally optimal when downstream noise is negatively correlated across pathways. The transitions can be most easily understood by considering the behavior of the optimal linear readout of the stimulus for each strategy. This readout is a *difference* of the total responses for ON-OFF pairs, while it is a (weighted) *sum* of the total responses for ON-ON pairs. Thus, ON-OFF pairs result in a subtraction of the downstream noise, while ON-ON pairs add the downstream noise. It is therefore favorable to encode with an ON-OFF pair when downstream noise is positively correlated and with an ON-ON pair when downstream noise is negatively correlated. Although the intuition is clearer for the linear readout, this picture also holds when MI is maximized (see S2 and S3 Figs).

The exact values of downstream noise variance and correlations at which the transition from ON-OFF to ON-ON strategies being optimal depends on the upstream and scaled Poisson noise. The strength *κ* adjusts the height of the transition boundaries: increasing *κ* tends to increase the range of downstream noise strength and correlations for which ON-OFF is optimal, while taking *κ* → 0 shifts the boundary curve down until the transition occurs at *ρ*_down_ = 0 for all *σ*_down_.

## Discussion

While the efficient coding hypothesis has been an important principle in understanding neural coding, our results demonstrate that proper interpretation of a neural circuit’s efficiency depends on the nature and location of noise; a nonlinearity that is efficient if noise enters at one location in the circuit may be inefficient if the noise actually enters in a different location.

Several previous studies have investigated how architecture or cell type impacts efficient coding strategies [20, 21, 35, 56–58]. While these models also include noise, assumptions about the location and strength of noise, as well as the allowed shapes of the nonlinearities, are more restrictive than our approach, and are not intended to systematically investigate the effects of disparate noise sources on coding strategies. Many similar questions can be answered using our model, and thus our work complements these studies by providing a broader, unifying picture of the interplay between noise and circuit architecture or cell types, while highlighting how different assumptions about noise could alter the conclusions or interpretation of previous work.

### Implications for efficient coding in biological circuits

Noise in neural circuits arises from a variety of sources, both internal and external to the nervous system (reviewed in [1]). Noise is present in sensory inputs, such as fluctuations in photon arrival rate at the retina, which follow Poisson statistics, or variability in odorant molecule arrival at olfactory receptors due to random diffusion and the turbulent nature of odor plumes. Noise also arises within the nervous system due to several biophysical processes, such as sensory transduction cascades, channel opening, synaptic vesicle release, and neurotransmitter diffusion.

Past work has focused on two complementary, but distinct aspects of neural coding: 1) how noise limits coding fidelity, and 2) how circuits should efficiently encode inputs in the presence of such noise. Much of the work to date has focused on the first aspect, investigating how noise places fundamental limits on information transfer and coding fidelity for fixed neural coding strategies (e.g., tuning curves) [2–5]. Examples include studying how noise correlations lead to ambiguous separation of neural responses [2] and which correlation structures maximally inhibit coding performance [5].

The second perspective dates back to the pioneering work of [6] and [7]. These early works primarily considered how efficient codes are affected by constraints on neural responses, such as limited dynamic range. Recent studies have built upon these foundational studies, investigating further questions such as how circuit architecture shapes optimal neural codes [20, 21, 35, 56–58]. However, this body of work has not systematically studied how efficient coding strategies depend on assumptions made about the nature of noise in a circuit.

Previous work has shown that the amount of noise in a circuit can qualitatively change optimal coding strategies [8, 59]. We also find that noise strength can be an important factor in determining efficient coding strategies. A 5- to 10-fold decrease in the signal-to-noise ratio produces dramatic qualitative changes in the optimal nonlinearities (Fig 2), and those changes depend on noise location. The SNR values used in our study correspond to a range of SNR values commonly observed in responses of neurons in early sensory systems [60, 61], suggesting that this result could be observed in biological circuits. Our analysis goes beyond considerations of noise strength to reveal how efficient coding strategies change depending on where noise arises in a circuit, showing that different noise sources often having competing effects. Other work in the context of decision making has similarly shown that the location of noise can impact the optimal architecture of a network, thus demonstrating that noise location in a circuit is important not only for signal transmission but also for computation [62]. Knowledge of both noise strength and where noise arises is therefore crucial for determining whether a neural circuit is encoding efficiently or not. Notably, even when the SNR of the circuit outputs is the same, the optimal nonlinearity can be very different depending on the location of the dominant noise source.

The locations of different noise sources have perhaps been most clearly elucidated in the retina. Several studies have investigated noise within the photoreceptors, and in some cases have even implicated certain elements within the transduction cascade [61, 63, 64]. Additional noise arises at the photoreceptor to bipolar cell synapse, where stochastic fluctuations in vesicle release obscure the signal [45, 65–67]. It has also been suggested that noise downstream of this synapse contributes a significant amount of the total noise observed in the ganglion cells, with some studies pointing to the bipolar cell to ganglion cell synapse specifically [26, 67].

Several pieces of evidence show that the relative contributions of different noise sources can change under different conditions as a circuit adapts. For example, in starlight or similar conditions, external noise due to variability in photon arrival dominates noise in rod photoreceptors and the downstream retinal circuitry [61, 68–70]. As light levels increase, noise in the circuits reading out the photoreceptor signals—particularly at the synapse between cone bipolar cells and ganglion cells—can play a more prominent role [26, 67]. Moreover, even in cases where the magnitude of a given noise source remains unchanged, adaptation can engage different nonlinearities throughout the circuit, shifting the location of the dominant nonlinearity and thereby effectively changing the location of the noise sources relative to the circuit nonlinearity. The fact that noise strength and nonlinearity location in neural circuits is subject to change under different conditions underscores the importance of understanding how these circuit features shape optimal encoding strategies.

In the retina, it has been observed that the nonlinearity at the cone bipolar to ganglion cell synapse can change dramatically depending on ambient illumination. Under daylight viewing conditions, this synapse exhibits strong rectification. Yet under dimmer viewing conditions, this synapse is nearly linear [42]. The functional role of this change is unclear, though the fact that noise sources are known to change under different levels of illumination points to a possible answer. If the dominant source of noise shifts from external sources to sources within downstream circuitry with increasing light level, as suggested by the evidence in [42], our results indicate that the circuit indeed ought to operate more nonlinearly at higher light levels. Furthermore, it is known that the strength of correlations not only varies between different types of retinal ganglion cells [71], but these correlations may be stimulus dependent [72, 73]. Based on our results for paired nonlinearities, we predict that types of neurons that receive highly correlated input will have nonlinearities with small overlap, while cells that receive uncorrelated input will have highly overlapping nonlinearities. Fully understanding this adaptation, and adaptations in other systems, will require further elucidation of the noise sources in the circuit.

### Reinterpretation of other efficient coding studies

Understanding how different aspects of circuit architecture shape efficient coding strategies has been a recent area of interest [20, 21, 35, 56–58]. However, a systematic study of the effects of noise was not the goal of these works, and so the properties of the noise in these studies has been limited, bound by specific assumptions on noise strength and location, and the allowed shapes of nonlinearities. As a result, while there is some overlap in the conclusions of these studies, the differences in assumptions about the noise and nonlinearities also lead to some apparent disagreement. Fortunately, we can investigate many similar questions within our model, and thereby complement the results of these previous studies and enrich our understanding of the role of circuit architecture and function. We briefly discuss the connections that other published studies have to the work presented here, focusing on studies with questions that can be most directly investigated as special cases of our model.

Early work by Laughlin suggested a simple solution for how a single neuron can maximize the amount of information transmitted: a neuron should utilize all response levels with equal frequency, thereby maximizing the response entropy [7]. Laughlin found that an interneuron in the compound eye of the blowfly transforms its inputs according to this principle. More recent work investigated nonlinearities in salamander and macaque retinal ganglion cells, predicting that optimal nonlinearities should be steep with moderate thresholds [35]. Experimental measurements of nonlinearities in ganglion cells were found to be near-optimal based on these predictions. Although both of these studies (along with many others) predict that neurons are efficiently encoding their inputs, assumptions about noise are not well-constrained by experiment. (In one case, the model assumes very low noise of equal magnitude for all output levels, while in the other all noise is at the level of the nonlinearity output.) As our work shows, one can arrive at different—even opposite—conclusions depending on where noise is assumed to enter the circuit. Without experimentally determining the sources of noise in each circuit, it is impossible to determine whether that circuit is performing optimally.

Going beyond single neurons or pathways, several recent studies have investigated the benefits of using multiple channels to encode stimuli and assigning different roles to each of those channels depending on circuit inputs. For example, Gjorgjieva and colleagues investigated when it is beneficial to encode inputs with multiple neurons of the same polarity versus encoding inputs with neurons of different polarity [56]. They conclude that ON-ON and ON-OFF circuits generally produce the same amount of mutual information, with ON-OFF circuits doing so more efficiently per spike. Our results provide a broader context in which we can interpret their findings, showing that when additive downstream noise (which was not included in their model) is anti-correlated, encoding with same polarity neurons can become a more favorable solution. Another recent study investigated under what conditions it is beneficial for multiple neurons of the same polarity to have the same threshold and when it is beneficial to split thresholds [21]. In particular, [21] find that nonlinearities split when the strength of upstream noise is weak. Our results are consistent with this finding and again broaden our understanding of why this splitting occurs: by incorporating correlations, we show that it is not simply the amount of noise that determines splitting, but the combination of noise strength and noise correlations. This identifies additional possibilities for testing these efficient coding predictions, by looking not just for cells that receive noisy input with similar magnitudes, but by looking for types of cells that receive correlated versus uncorrelated input and determining the degree of overlap of their nonlinearities.

### Conclusion

We find that even in relatively simple circuit models, assumptions about the location and strength of multiple noise sources in neural circuits strongly impact conclusions about optimal encoding. In particular, different relative strengths of noise upstream, downstream, or associated with nonlinear processing of signals yield different optimal coding strategies, even if the overall signal-to-noise ratio is the same. Furthermore, correlations between noise sources across multiple channels alter the degree to which optimal channels encode overlapping portions of the signal distribution, as well as the overall polarity of the channels. On the other hand, different combinations of noise sources can also yield very similar nonlinearities. Consequently, measurements of noise at various locations in neural circuits are necessary to verify or refute ideas about efficient coding and to more broadly understand the strategies by which neurons mitigate the effects of unwanted variability in neural computations.

## Methods

### Model details

Our model is schematized in Fig 1. Biophysical interpretation is discussed in detail in the Results and Discussion. We model the input to the circuit as a signal or stimulus *s* that comes from a distribution of possible inputs within a short integration time window, and hence is a random variable in our model. Before this input can be encoded by the circuit, it is corrupted by noise *η*, which we also take to be a random variable. The circuit then encodes total signal *s* + *η* by nonlinearly transforming it, *f*(*s* + *η*). This transformed signal sets the mean of a variable circuit response. That is, the circuit does not respond deterministically, but stochastically. We do not take this stochastic response to be spiking, due to the fact that spike generation has been shown to be repeatable, attributing variability in spiking to other sources [22–24]. Instead, inspired by quantal neurotransmitter release, which results in post-synaptic potentials of integer multiples of a fixed minimum size, we model the stochastic response as a scaled Poisson distribution: responses come in integer multiples of a minimum non-zero response size *κ*, with an overall mean response *f*(*s* + *η*), conditioned on the total input, *s* + *η*. This stochastic response is then corrupted by downstream noise *ζ*, which we also take to be a random variable. The total response *r* of a single-path circuit is thus

$$\begin{array}{c} r=\kappa m+\zeta , \end{array}$$ (2)

where *m* is a Poisson-distributed random variable with mean *κ*^ − 1^ *f*(*s* + *η*), such that the mean of *κm* is *f*(*s* + *η*). Our circuit model thus has three sources of intrinsic variability: the additive noise sources (*η* and *ζ*) and the stochastic scaled-Poisson response.

We assume the statistics of the signal and noise are held fixed over a time window long enough that the circuit can adapt its nonlinearity to the full distribution of signal and noise. That is, in a small integration time window *Δt*, the channel receives a draw from the signal and noise distributions to produce a response. Thus, we model the signal *s* and noises *η*, *ζ*, and the scaled Poisson responses as random variables rather than stochastic processes.

In this work, we assume the distribution of possible inputs to be Gaussian with fixed variance $\sigma_{s}^{2}$; without loss of generality we can take the mean to be zero (i.e., the signal represents variations relative to a mean background). We assume the upstream and downstream noise to be Gaussian with mean 0 and variances $\sigma_{\text{up}}^{2}$ and $\sigma_{\text{down}}^{2}$, respectively. The assumption of Gaussian distributions for the input and noise is not a restriction of the model, but a choice we make to simplify our analyses and because we expect physiologically relevant noise sources to share many of the properties of a Gaussian distribution. Even in cases where the input distribution is not Gaussian, pre-processing of inputs can remove heavy tails and lead to more Gaussian-like input distributions. It has been shown that stimulus filtering in the retina indeed has this effect [74].

An additional scenario to consider is the possibility that the signal properties, such as the variances, could themselves be random. We might then wonder how this would impact the predicted nonlinearities. As a “trial” of our model is a single draw from the stimulus and noise distributions, there is no well-defined variance on a *single* trial. A changing variance on every trial would be equivalent to starting with a broader noise distribution of fixed variance. We can thus interpret the stimulus distribution we use in the study to be the effective distribution after trial-by-trial variations in variance have already been taken into account. The results for a signal of constant variance can thus be adapted, qualitatively, to the case of random trial-by-trial variance by increasing the stimulus variance in order to mimic the impact that trial-by-trial changes in variance have on the shape of the nonlinearity.

### Two methods for determining the optimal nonlinearities

In order to understand how noise properties and location impact efficient coding strategies, we seek the nonlinearity that best encoded the input distribution for a variety of noise conditions. We primarily consider the mean squared error (MSE) of a linear estimator of the stimulus, as outlined below, as our criterion of optimality. This is not the only possible optimality criterion, so to check the effects that other criteria might have, we also consider maximizing the mutual information (MI) between stimulus and response. MI provides a measure of coding fidelity that is free from assumptions about how information is read out from responses. However, MI is difficult to evaluate analytically for all but the simplest models. Indeed, for our model, deriving exact analytic equations for the optimal nonlinearities using MI is intractable. We turn to simulations in this case.

We determine the nonlinearities obtained by minimizing the MSE using two complementary methods. First, we take variational derivatives of the MSE with respect to the nonlinearities themselves to derive a set of exact equations for the optimal nonlinearities, free from any assumptions about their shape or functional form, as described below. The only constraints we apply are that the nonlinearity must be non-negative and saturate at a value of 1. (The choice of saturation level is arbitrary and does not affect the results.) Applying such constraints are non-trivial—in most variational problems constraints enforce an equality, but in our method we are enforcing an inequality, discussed in the next section. Using this analytic approach, we minimize the assumptions we make about the nonlinearities and obtain insights into the behavior of the model that are otherwise inaccessible.

**Fig 8:**
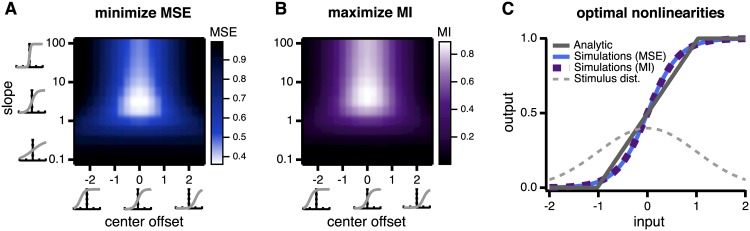
Complementary methods for determining the optimal nonlinearity. **A:** We use simulations to find the sigmoidal nonlinearity that minimizes the mean squared error (MSE) of a linear readout of the stimulus. We sweep over multiple possible slope and offset combinations to find the optimal nonlinearity. MSE is given in units of the stimulus variance, $\sigma_{s}^{2}$. **B:** Using the same method as **A**, we maximize the mutual information (MI) between stimulus and responses. MI is given in units of bits. **C:** Optimal sigmoidal nonlinearities (blue and purple curves) found from simulations versus the optimal nonlinearity determined by solving the model analytically (gray curve). The analytical solution is determined non-parametrically, and was not chosen to be piecewise linear. All nonlinearities are qualitatively similar, regardless of the criterion for optimality or constraints on the functional form of the nonlinearity.

Second, we parametrize the nonlinearities as sigmoidal or piecewise linear curves with two parameters that control the slope and offset. We simulate the model, sweeping over the slope and offset parameters (Fig 8A) until we find the parameter set that minimizes the MSE of the linear readout. This parametric approach makes strong assumptions about the form of the nonlinearity but also has distinct advantages. Simulations allow us to test to what extent our conclusions about the shape (i.e., slope and offset) of the optimal nonlinearity depend on its specific functional form. For example, we find from our analytical calculations that optimal nonlinearities are roughly piecewise linear (Fig 8C), but one might expect biophysical constraints to restrict neurons to having smooth nonlinearities. For this reason, we also test sigmoidal shaped nonlinearities, a smooth approximation of the piecewise linear solutions that emerge from the nonparametric analytical approach, and use simulations to find the optimal parameters. We find the results with sigmoidal nonlinearities qualitatively very similar to the analytical solution (Fig 8C).

Parametric simulations have the additional advantage of allowing tests of more complex criteria for optimality than the MSE, such as maximizing the mutual information (MI) between the stimulus and responses, which we cannot compute analytically. Using simulations with parametrized nonlinearities, we are able to find the nonlinearity that maximizes MI (Fig 8B). We have verified that optimal nonlinearities found by maximizing MI are qualitatively similar to those found by minimizing the MSE of a linear readout (Fig 8C shows one example). For simplicity, throughout the main text of this paper we focus on results for minimizing MSE, but present results from maximizing MI in a few cases for comparison.

### Variational approach

#### Single pathway

We first evaluate how well a single pathway encodes the signal *s* using the mean squared error (MSE) between the true signal *s* and an estimate of the signal, *s*_est_, computed from the responses:

$$\begin{array}{c} \chi^{2}={\left\langle {\left(s-s_{\text{est}}\right)}^{2}\right\rangle }_{s,r}, \end{array}$$ (3)

where the average is taken over all possible inputs and all possible responses (or, equivalently, all possible inputs and all possible configurations of intrinsic circuit variability). We choose as our signal-estimator a linear function of the response:

$$\begin{array}{c} s_{\text{est}}=D_{0}+Dr. \end{array}$$ (4)

The parameter *D* is a “decoding weight” that sets the scale between the signal estimate and the responses. It can be shown that minimizing *χ*^2^ with respect to the parameter *D*_0_ yields *D*_0_ = −*D*〈*r*〉, so the estimator may be written

$$\begin{array}{c} s_{\text{est}}=D\left(r-\left\langle r\right\rangle \right). \end{array}$$

For the linear estimator, the expression for the MSE yields

$$\begin{array}{c} \chi^{2}=\sigma_{s}^{2}-2D\left\langle sf\left(s+\eta \right)\right\rangle +D^{2}\left(\kappa \left\langle f\left(s+\eta \right)\right\rangle +\text{var}\left[f\left(s+\eta \right)\right]+\sigma_{\text{down}}^{2}\right), \end{array}$$ (5)

where the averages 〈…〉 are taken over the stimulus and noise distributions: *s*, *η*, *ζ* (averages over the scaled-Poisson variable have been evaluated completely). This expression for the MSE is a function of the decoding weight *D* and a functional of the nonlinearity *f*(*z*). To find the most efficient coding strategy for a fixed set of noise parameters $\sigma_{\text{up}}^{2}$, *κ*, and $\sigma_{\text{down}}^{2}$, we must minimize *χ*^2^ with respect to *both* the decoding weight *D* (which gives the “optimal linear estimator”) and the nonlinearity *f*(*z*).

We can minimize *χ*^2^ with respect to the decoding weight *D* by taking a partial derivative and setting it equal to zero, yielding

$$\begin{array}{c} D\left(\kappa \left\langle f\left(s+\eta \right)\right\rangle +\text{var}\left[f\left(s+\eta \right)\right]+\sigma_{\text{down}}^{2}\right)=\left\langle sf\left(s+\eta \right)\right\rangle . \end{array}$$ (6)

To determine the optimal nonlinearity, subject to the constraints 0 ≤ *f*(*z*) ≤ 1, we take a variational derivative of the MSE with respect to the nonlinearity itself. The constraints can be formally applied using a continuous version of the Karush–Kuhn–Tucker (KKT) conditions, an extension of the method of Lagrange multipliers to inequality constraints. This allows us to free ourselves from *all* assumptions about the shape of the nonlinearity outside of the limited dynamic range we impose. For Gaussian stimuli and upstream noise of respective variances $\sigma_{s}^{2}$ and $\sigma_{\text{up}}^{2}$, the resulting equation for *f*(*z*) is

$$\begin{array}{c} f\left(z\right)=\Xi \left(\frac{\sigma_{s}^{2}}{\sigma_{s}^{2}+\sigma_{\text{up}}^{2}}\frac{z}{D}-\frac{\kappa }{2}+\left\langle f\left(s+\eta \right)\right\rangle \right), \end{array}$$ (7)

where *Ξ*(*x*) = 0 if *x* < 0, 1 if *x* > 1, and *x* if 0 ≤ *x* ≤ 1; this imposes the constraints on the solution. From Eq (7), we read off that the optimal single-pathway nonlinearity is a piecewise linear function (i.e., the nonlinearity is of the form *mz* + *b* for *z*_0_ < *z* < *z*_1_, and is 0 for *z* < *z*_0_ and 1 for *z* > *z*_1_). It is important to reiterate that, other than the limited dynamic range, we did not impose any assumptions (such as monotonicity) on the nonlinearity. All that remains is to self-consistently determine the constants *D* and 〈*f*(*s* + *η*)〉. Rather than solve directly for these constants, it is convenient to write the nonlinearity as

$$\begin{array}{c} f\left(z\right)=\Xi \left(\frac{z-z_{0}}{z_{1}-z_{0}}\right), \end{array}$$ (8)

and solve for the constants *z*_0_ and *z*_1_, which are defined by $f(z_{0}^{+})=0$ and $f(z_{1}^{-})=1$. This yields the relations $z_{1}-z_{0}=D\left(1+\sigma_{\text{up}}^{2}/\sigma_{s}^{2}\right)$ and *z*_0_ = (*z*_1_ − *z*_0_)(*κ*/2 − 〈*f*(*s* + *η*)〉). Using these relations and Eq (8) to compute the expectations appearing in Eq (6) yields a set of transcendental equations for *z*_1_ and *z*_0_ that can be solved numerically for any values of the noise parameters $\sigma_{s}^{2}$, $\sigma_{\text{up}}^{2}$, *κ* and $\sigma_{\text{down}}^{2}$. The expectation integrals 〈…〉 over *s* and *η* can be non-dimensionalized by a change of variables that shows that $z_{1}=\sqrt{\sigma_{s}^{2}+\sigma_{\text{up}}^{2}}x_{1}$ and $z_{0}=\sqrt{\sigma_{s}^{2}+\sigma_{\text{up}}^{2}}x_{0}$, for rescaled inputs *x*_1_ and *x*_0_ that depend only on *κ* and $\sigma_{\text{down}}^{2}$. Following this result, we set $z=\sqrt{\sigma_{s}^{2}+\sigma_{\text{up}}^{2}}x$ in Eq (7). The resulting coefficient of *x* is $\sigma_{s}^{2}/\sqrt{\sigma_{s}^{2}+\sigma_{\text{up}}^{2}}/D$. Non-dimensionalizing the expectation values in Eq (6) reveals that this parameter combination only depends on the non-dimensionalized expectation integrals, and so *f*(*z*) normalizes the input *z* by the standard deviation of *z*. As a result, the rescaled nonlinearity $f\left(\sqrt{\sigma_{s}^{2}+\sigma_{\text{up}}^{2}}x\right)=\tilde{f}\left(x\right)$ is independent of $\sigma_{s}^{2}$ and $\sigma_{\text{up}}^{2}$.

#### Two pathways

Our model of a two-pathway circuit encoding a common signal input builds on two copies of the single-pathway circuit. Both paths receive the same input signal *s*, which is then corrupted by noise upstream of the encoding nonlinearity. The corrupting noise is not the same in each pathway, but may be correlated. Thus, the total input to pathway 1 is *s* + *η*_1_ and the total input to pathway 2 is *s* + *η*_2_. Each pathway then encodes its input by nonlinearly transforming it, *f*_1_(*s* + *η*_1_) and *f*_2_(*s* + *η*_2_). The transformed signals set the mean of the scaled-Poisson noise in each path. The stochastic responses of each path are conditionally independent of each other. That is, correlations in the stochastic responses are only due to correlations in the inputs to the two pathways, not due to any intrinsic correlation. Finally, these stochastic responses are each corrupted by noise downstream of the encoding nonlinearity. The noise is not the same in each pathway, but may be correlated across pathways.

The responses for each pathway may be formulated mathematically as

$$r_{1}=\kappa m_{1}+\zeta_{1},$$ (9)

$$r_{2}=\kappa m_{2}+\zeta_{2},$$ (10)

where *m*_1_ and *m*_2_ are Poisson-distributed integers with (conditional) means *κ*^−1^ *f*_1_(*s* + *η*_1_) and *κ*^−1^ *f*_2_(*s* + *η*_2_), respectively. As in the single-pathway model, we assume there is a distribution of inputs *s* that we model as a zero-mean Gaussian with variance $\sigma_{s}^{2}$. As the upstream noise is correlated, *η*_1_ and *η*_2_ follow a joint-distribution that we take to be a zero-mean Gaussian with equal variances $\sigma_{\text{up}}^{2}$ in each pathway and correlation coefficient *ρ*_up_. Similarly, we model the downstream noise as a zero-mean bivariate Gaussian with variance $\sigma_{\text{down}}^{2}$ in both pathways and correlation coefficient *ρ*_down_. The rationales for choosing Gaussian distributions are the same as in the single-pathway model. The choice of equal upstream or downstream noise variance in each channel simplifies the analysis; unequal variances are tractable but offer little additional insight, so we do not discuss this case in this work.

As in the single-path case, we determine the optimal choices of nonlinearities by minimizing the mean-squared-error between the input to the circuit and an estimate computed from the responses of each pathway. We estimate the signal using a weighted sum of the responses,

$$\begin{array}{c} s_{\text{est}}=D_{1}\left(r_{1}-\left\langle r_{1}\right\rangle \right)+D_{2}\left(r_{2}-\left\langle r_{2}\right\rangle \right). \end{array}$$ (11)

(The constant shift *D*_0_ has been decomposed into the optimal choice, *D*_0_ = −*D*_1_〈*r*_1_〉 − *D*_2_〈*r*_2_〉.) The MSE for this choice of decoder works out to

$$\begin{array}{ccc} \chi^{2} & = & \sigma_{s}^{2}-2D_{1}\left\langle sf_{1}\left(s+\eta_{1}\right)\right\rangle -2D_{2}\left\langle sf_{2}\left(s+\eta_{2}\right)\right\rangle \\ & & +D_{1}^{2}\left(\kappa \left\langle f_{1}\left(s+\eta_{1}\right)\right\rangle +\text{var}\left[f_{1}\left(s+\eta_{1}\right)\right]+\sigma_{\text{down}}^{2}\right)\\ & & +D_{2}^{2}\left(\kappa \left\langle f_{2}\left(s+\eta_{2}\right)\right\rangle +\text{var}\left[f_{2}\left(s+\eta_{2}\right)\right]+\sigma_{\text{down}}^{2}\right)\\ & & +2D_{1}D_{2}\left(\text{cov}\left[f_{1}\left(s+\eta_{1}\right),f_{2}\left(s+\eta_{2}\right)\right]+\sigma_{\text{down}}^{2}\rho_{\text{down}}\right); \end{array}$$ (12)

averages in this expression are taken over the stimulus *s*, and the joint distributions of *η*_1_ and *η*_2_; averages over *ζ*_1_ and *ζ*_2_ have been performed, yielding terms depending on the variance ($\sigma_{\text{down}}^{2}$) and correlation (*ρ*_down_) of the downstream noise. Taking regular derivatives with respect to the decoding weights *D*_1_ and *D*_2_ and variational derivatives with respect to *f*_1_(*z*) and *f*_2_(*z*) yields a coupled set of integral equations for the optimal weights and nonlinearities (subject again to the constraints 0 ≤ *f*_1_(*z*) ≤ 1 and 0 ≤ *f*_2_(*z*) ≤ 1). To simplify the resulting equations, we present them here in rescaled form, defining the rescaled nonlinearities ${\tilde{f}}_{1}\left(x\right)=f_{1}\left(\sqrt{\sigma_{s}^{2}+\sigma_{\text{up}}^{2}}x\right)$ and ${\tilde{f}}_{2}\left(x\right)=f_{2}\left(\sqrt{\sigma_{s}^{2}+\sigma_{\text{up}}^{2}}x\right)$, as well as the rescaled decoder weights ${\tilde{D}}_{1}=\sqrt{\sigma_{s}^{2}+\sigma_{\text{up}}^{2}}D_{1}/\sigma_{s}^{2}$ and ${\tilde{D}}_{2}=\sqrt{\sigma_{s}^{2}+\sigma_{\text{up}}^{2}}D_{2}/\sigma_{s}^{2}$. This reflects that, as in the single-pathway case, the optimal nonlinearities rescale inputs by the total input standard deviation $\sqrt{\sigma_{s}^{2}+\sigma_{\text{up}}^{2}}$. The equations for the rescaled decoder weights and nonlinearities are

$$\begin{array}{c} {\tilde{D}}_{1}\left(\kappa \left\langle {\tilde{f}}_{1}\right\rangle +\text{var}\left[{\tilde{f}}_{1}\right]+\sigma_{\text{down}}^{2}\right)+{\tilde{D}}_{2}\left(\text{cov}\left[{\tilde{f}}_{1},{\tilde{f}}_{2}\right]+\sigma_{\text{down}}^{2}\rho_{\text{down}}\right)=\left\langle y{\tilde{f}}_{1}\right\rangle , \end{array}$$ (13)

$$\begin{array}{c} {\tilde{D}}_{2}\left(\kappa \left\langle {\tilde{f}}_{2}\right\rangle +\text{var}\left[{\tilde{f}}_{2}\right]+\sigma_{\text{down}}^{2}\right)+{\tilde{D}}_{1}\left(\text{cov}\left[{\tilde{f}}_{2},{\tilde{f}}_{1}\right]+\sigma_{\text{down}}^{2}\rho_{\text{down}}\right)=\left\langle y{\tilde{f}}_{2}\right\rangle , \end{array}$$ (14)

$$\begin{array}{c} {\tilde{f}}_{1}\left(x\right)=\Xi \left(\frac{x}{{\tilde{D}}_{1}}-\frac{\kappa }{2}+\left\langle {\tilde{f}}_{1}\right\rangle +\frac{{\tilde{D}}_{2}}{{\tilde{D}}_{1}}\left[\left\langle {\tilde{f}}_{2}\right\rangle -\int_{-\infty }^{\infty }dy\;\frac{e^{-\frac{1}{2}\frac{{\left(y-\rho_{\text{eff}}x\right)}^{2}}{1-\rho_{\text{eff}}^{2}}}}{\sqrt{2\pi (1-\rho_{\text{eff}}^{2})}}{\tilde{f}}_{2}\left(y\right)\right]\right), \end{array}$$ (15)

$$\begin{array}{c} {\tilde{f}}_{2}\left(x\right)=\Xi \left(\frac{x}{{\tilde{D}}_{2}}-\frac{\kappa }{2}+\left\langle {\tilde{f}}_{2}\right\rangle +\frac{{\tilde{D}}_{1}}{{\tilde{D}}_{2}}\left[\left\langle {\tilde{f}}_{1}\right\rangle -\int_{-\infty }^{\infty }dy\;\frac{e^{-\frac{1}{2}\frac{{\left(y-\rho_{\text{eff}}x\right)}^{2}}{1-\rho_{\text{eff}}^{2}}}}{\sqrt{2\pi (1-\rho_{\text{eff}}^{2})}}{\tilde{f}}_{1}\left(y\right)\right]\right), \end{array}$$ (16)

where again Ξ(*x*) = 0 if *x* < 0, 1 if *x* > 1, and *x* if 0 ≤ *x* ≤ 1. Here, the averages 〈…〉 involving single nonlinearities are integrals with respect to a standard normal distribution weight, $\exp\left(-y^{2}/2\right)/\sqrt{2\pi }$; e.g.,

$$\begin{array}{c} \left\langle y\tilde{f}\right\rangle =\int_{-\infty }^{\infty }dy\;\frac{e^{-\frac{y^{2}}{2}}}{\sqrt{2\pi }}y\tilde{f}\left(y\right). \end{array}$$

The covariance between ${\tilde{f}}_{1}\left(x\right)$ and ${\tilde{f}}_{2}\left(x\right)$, $\text{cov}[{\tilde{f}}_{1},{\tilde{f}}_{2}]$, is defined as

$$\begin{array}{c} \text{cov}\left[{\tilde{f}}_{1},{\tilde{f}}_{2}\right]=\left\langle {\tilde{f}}_{1}{\tilde{f}}_{2}\right\rangle -\left\langle {\tilde{f}}_{1}\right\rangle \left\langle {\tilde{f}}_{2}\right\rangle , \end{array}$$

where

$$\begin{array}{c} \left\langle {\tilde{f}}_{1}{\tilde{f}}_{2}\right\rangle =\int_{-\infty }^{\infty }dy_{1}dy_{2}\;{\tilde{f}}_{1}\left(y_{1}\right)\frac{e^{-\frac{1}{2}\frac{y_{1}^{2}+y_{2}^{2}-2\rho_{\text{eff}}y_{1}y_{2}}{1-\rho_{\text{eff}}^{2}}}}{2\pi \sqrt{1-\rho_{\text{eff}}^{2}}}{\tilde{f}}_{2}\left(y_{2}\right). \end{array}$$ (17)

The effective upstream noise correlation coefficient is defined by Eq (1) in the main text, and represents the total effective correlation between the inputs *s* + *η*_1_ and *s* + *η*_2_; i.e., the perfectly correlated inputs to the two pathways (the two copies of *s*) are corrupted by noise, thereby reducing the overall correlation of the inputs to the two channels.

Unlike the single-pathway case, the equations for the paired-pathway nonlinearities are coupled nonlinear integral equations that must, in general, be solved numerically. Two special cases can be solved by hand: *ρ*_eff_ = 0, in which the inputs to two pathways are effectively uncorrelated (requiring large, negatively correlated upstream noise) and the equations decouple, yielding two copies of the single-pathway nonlinearity. The second case is *ρ*_eff_ = 1, for which the inputs to the two channels are perfectly correlated (either due to the lack of upstream noise or because upstream noise is perfectly correlated between the two channels). In this case, the Gaussian integral kernels in Eqs (15) and (16) reduce to delta functions, yielding two coupled functional equations for the nonlinearities that can be solved to show the optimal nonlinearities are piecewise linear. The resulting pair of nonlinearities may have the same polarity of responses to stimuli, which we call “ON-ON” or “OFF-OFF” pairs (by analogy to ON and OFF cells in the retina) or opposite polarity responses to stimuli, which we dub “ON-OFF” or “OFF-ON” pairs.

#### Numerical solution of the coupled integral equations for the paired nonlinearities

To solve the paired nonlinearities in general, we formulate Eqs (13)–(16) as fixed points of the set of iterated mappings

$$\begin{array}{c} {\tilde{D}}_{1}^{\left(n+1\right)}=\frac{\left(\kappa \left\langle {\tilde{f}}_{2}^{\left(n\right)}\right\rangle +\text{var}\left[{\tilde{f}}_{2}^{\left(n\right)}\right]+\sigma_{\text{down}}^{2}\right)\left\langle y{\tilde{f}}_{1}^{\left(n\right)}\right\rangle -\left(\text{cov}\left[{\tilde{f}}_{1}^{\left(n\right)},{\tilde{f}}_{2}^{\left(n\right)}\right]+\sigma_{\text{down}}^{2}\rho_{\text{down}}\right)\left\langle y{\tilde{f}}_{2}^{\left(n\right)}\right\rangle }{\prod_{i=1}^{2}\left(\kappa \left\langle {\tilde{f}}_{i}^{\left(n\right)}\right\rangle +\text{var}\left[{\tilde{f}}_{i}^{\left(n\right)}\right]+\sigma_{\text{down}}^{2}\right)-{\left(\text{cov}\left[{\tilde{f}}_{1}^{\left(n\right)},{\tilde{f}}_{2}^{\left(n\right)}\right]+\sigma_{\text{out}}^{2}\rho_{\text{down}}\right)}^{2}}, \end{array}$$ (18)

$$\begin{array}{c} {\tilde{D}}_{2}^{\left(n+1\right)}=\frac{\left(\kappa \left\langle {\tilde{f}}_{1}^{\left(n\right)}\right\rangle +\text{var}\left[{\tilde{f}}_{1}^{\left(n\right)}\right]+\sigma_{\text{down}}^{2}\right)\left\langle y{\tilde{f}}_{2}^{\left(n\right)}\right\rangle -\left(\text{cov}\left[{\tilde{f}}_{1}^{\left(n\right)},{\tilde{f}}_{2}^{\left(n\right)}\right]+\sigma_{\text{out}}^{2}\rho_{\text{down}}\right)\left\langle y{\tilde{f}}_{1}^{\left(n\right)}\right\rangle }{\prod_{i=1}^{2}\left(\kappa \left\langle {\tilde{f}}_{i}^{\left(n\right)}\right\rangle +\text{var}\left[{\tilde{f}}_{i}^{\left(n\right)}\right]+\sigma_{\text{down}}^{2}\right)-{\left(\text{cov}\left[{\tilde{f}}_{1}^{\left(n\right)},{\tilde{f}}_{2}^{\left(n\right)}\right]+\sigma_{\text{down}}^{2}\rho_{\text{down}}\right)}^{2}}, \end{array}$$ (19)

$$\begin{array}{c} {\tilde{f}}_{1}^{\left(n+1\right)}\left(x\right)=\Xi \left(\frac{x}{{\tilde{D}}_{1}^{\left(n\right)}}-\frac{\kappa }{2}+\left\langle {\tilde{f}}_{1}^{\left(n\right)}\right\rangle +\frac{{\tilde{D}}_{2}^{\left(n\right)}}{{\tilde{D}}_{1}^{\left(n\right)}}\left[\left\langle {\tilde{f}}_{2}^{\left(n\right)}\right\rangle -\int_{-\infty }^{\infty }\;dy\;\frac{e^{-\frac{y^{2}}{2}}}{\sqrt{2\pi }}{\tilde{f}}_{2}^{\left(n\right)}\left(\sqrt{1-\rho_{\text{eff}}^{2}}y+\rho_{\text{eff}}x\right)\right]\right), \end{array}$$ (20)

$$\begin{array}{c} {\tilde{f}}_{2}^{\left(n+1\right)}\left(x\right)=\Xi \left(\frac{x}{{\tilde{D}}_{2}^{\left(n\right)}}-\frac{\kappa }{2}+\left\langle {\tilde{f}}_{2}^{\left(n\right)}\right\rangle +\frac{{\tilde{D}}_{1}^{\left(n\right)}}{{\tilde{D}}_{2}^{\left(n\right)}}\left[\left\langle {\tilde{f}}_{1}^{\left(n\right)}\right\rangle -\int_{-\infty }^{\infty }\;dy\;\frac{e^{-\frac{y^{2}}{2}}}{\sqrt{2\pi }}{\tilde{f}}_{1}^{\left(n\right)}\left(\sqrt{1-\rho_{\text{eff}}^{2}}y+\rho_{\text{eff}}x\right)\right]\right). \end{array}$$ (21)

We formally solved the linear set of eqs (13) and (14) for the rescaled decoding weights to write down Eqs (18) and (19). We have also changed variables in the integrals to move all dependence on *ρ*_eff_ into the nonlinearities. This is necessary for numerical stability when *ρ*_eff_ is close to 1.

Though we have written the above equations in rescaled form for notational simplicity, the code was written in the original, unrescaled form, so we revert to that notation when describing the algorithm below.

The idea of this set of iterative mappings is to make initial guesses for $D_{1}^{\left(0\right)}$, $D_{2}^{\left(0\right)}$, $f_{1}^{\left(0\right)}\left(z\right)$, and $f_{2}^{\left(0\right)}\left(z\right)$, and use Eqs (18)–(21) to update the guesses until

$$\begin{array}{c} \left|D_{i}^{\left(n+1\right)}-D_{i}^{\left(n\right)}\right|<\epsilon , \end{array}$$

$$\begin{array}{c} \left|f_{i}^{\left(n+1\right)}\left(z\right)-f_{i}^{\left(n\right)}\left(z\right)\right|<\epsilon , \end{array}$$

for an absolute tolerance *ϵ* = 10^−4^, for every point *z*. This is a form of Picard iteration for finding the fixed points of iterated maps. It is difficult to prove convergence of this scheme, so we resort to solving the equations for multiple initial guesses and showing that they consistently converge to the same solutions. ON-OFF solutions can be found by making initial guess *D*_1_ > 0, *D*_2_ < 0, while ON-ON solutions can be found by making initial guess *D*_1_ > 0, *D*_2_ > 0. Our initial guesses are Gaussian random variables centered at ±1, with a standard deviation of 0.5.

Eqs (15) and (16) constrain the form of the nonlinearities, which allows us to consider a restricted set of initial guesses. Because the integral kernel has unit area and the nonlinearities are restricted to the range [0, 1], the integral term in Eqs (20) and (21) is also within the range [0, 1]. For initial guesses for the nonlinearities, we discretize the input values *z*, and for each input value we choose a uniform random value for the integral term in Eqs (20) and (21). The initial guess is also thresholded so that it satisfies the constraint $0\leq f_{i}^{\left(0\right)}\left(z\right)\leq 1$.

For each class of ON-OFF or ON-ON initial conditions, we use 20 different initial random seeds. The initial conditions for different seeds generally converge to the same solutions. Some rare exceptions occur: occasionally, an ON-ON or ON-OFF initial guess may find the opposite class of solution. For parameter regimes in which the MSE of identical and non-identical ON-ON solutions is very close, ON-ON guesses may result in either solution.

During the iterative computations, the discretized nonlinearities are fit with splines for use in numerically computing the various integrals. Single variable integrals are performed using a Gauss-Legendre quadrature scheme, while double-integrals are evaluated using Monte Carlo sampling.

For the parameter values used in the numerical solution of the integral equations, see the “Parameter values used in figures” section, below.

### Model simulations

Analytic calculations allow us to exactly determine the nonlinearities that minimize the MSE of a linear readout, without making any assumptions about the shape of the nonlinearity. However, it is possible that certain physiological properties might constrain the shape of the nonlinearity (to be smooth, for example). It is also possible that another criterion for optimality (instead of minimizing MSE of a linear readout) might yield different results. To test these possibilities, we turned to simulations.

#### Minimizing MSE for different nonlinearity shapes

We first tested the dependence of our results on the shape of the nonlinearity. Throughout the paper, we show results for logistic nonlinearities of the form:

$$\begin{array}{c} f\left(z\right)=\frac{1}{1+\exp(-\nu (z-\phi ))} \end{array}$$ (22)

where *ν* is a slope parameter and *ϕ* is an offset parameter. (Remember that we constrain the range of our nonlinearities to be between 0 and 1 to mimic physiological constraints that result in thresholding and saturating nonlinearities.) This particular form was chosen due to its smoothness (in contrast to the optimal nonlinearities found from analytic calculations) and because it can be characterized by just two parameters. For a given set of noise conditions, we drew randomly from the stimulus distribution and simulated responses that would be produced by a nonlinearity with slope parameter *ν* and offset *ϕ*. We then found the decoding weight(s) *D*, estimated the stimulus, and calculated the MSE. The number of draws required from the stimulus distribution to produce an accurate estimate varied widely depending on the noise parameters (with noisier conditions requiring more draws to accurately estimate the MSE). We generally used about 10 million draws from the stimulus distribution and averaged the results of 5–10 repetitions of this procedure to obtain our results. Comparison with analytical calculations verified the accuracy of our estimates. By completing this procedure for a number of different possible parameters for the nonlinearity, sweeping over a broad range of slope and offset values, we determined the parameters that minimized the MSE. These results are shown throughout the paper for comparison to the analytic solutions. The two classes are in broad agreement.

#### Maximizing mutual information

Simulations also allowed us to test the dependence of our results on the criterion for optimality. As a second criterion, we chose to maximize the mutual information:

$$\begin{array}{c} MI\left(S;R\right)=H\left(R\right)-{\left\langle H\left(R|S\right)\right\rangle }_{S} \end{array}$$ (23)

where *S* denotes the stimulus and *R* denotes the response. The (differential) entropy *H* is given by:

$$\begin{array}{c} H\left(X\right)=-\int_{-\infty }^{\infty }dx\;p\left(x\right)\log_{2}p\left(x\right) \end{array}$$ (24)

where *p*(*x*) denotes the probability density function of *x*. In order to estimate entropy, we used the binless estimator outlined by Victor in [75]. Briefly, this strategy relies on calculating nearest neighbor distances between points to estimate the distribution *p*(*x*); shorter distances indicate a higher density of points and greater *p*(*x*). The binless entropy estimate is given by:

$$\begin{array}{c} H_{\text{diff}}\left(X\right)\approx \log_{2}\left[\frac{S_{d}\left(M-1\right)}{d}\right]+\frac{\gamma }{\ln\left(2\right)}+\frac{d}{M}\sum_{j=1}^{M}\log_{2}\left(\lambda_{j}\right) \end{array}$$ (25)

*d* is the dimensionality of the distribution for which entropy is being estimated (in our case, the number of pathways), *M* is the number of samples, and *λ*_*j*_ is the Euclidean distance to the nearest neighbor of sample *j*. *S*_*d*_ is the area of a unit *d*-dimensional spherical surface (*S*_1_ = 2, *S*_2_ = 2*π*). *γ* is the Euler-Mascheroni constant. See [75] for further details. As with estimating the MSE, the number of draws from the stimulus distribution required for convergence was based on the value of the noise parameters. Generally, about 10,000 draws from the stimulus distribution were taken to estimate *H*(*R*). For each of those stimulus values, about 10,000–100,000 responses were simulated (on different “trials,” the same stimulus presented repeatedly yields different responses due to noise) to estimate *H*(*R*|*S*). Results of about 10 repetitions were averaged to obtain the final MI estimate.

The binless method requires that no two samples be identical, which can pose problems in certain conditions. For example, if *κ* is nonzero such that the output of the nonlinearity is discretized into a certain number of bins and downstream noise is zero or very small, many responses are likely to be identical to numerical precision. In these cases, a more standard binned method was used to estimate entropy:

$$\begin{array}{c} H_{\text{diff}}\left(X\right)\approx -\sum_{j=1}^{M}p\left(x_{j}\right)\log_{2}\left(\frac{p(x_{j})}{w}\right) \end{array}$$ (26)

where *w* is the bin width. Similar numbers of draws from the stimulus distribution were used as were used with the binless estimator. Generally, MI estimates converged when ∼50 bins were used.

Several test cases were used to verify that the binless and binned approaches yielded consistent estimates of the mutual information. MI estimates were additionally verified by comparing the estimates produced by these methods to particular cases in which the response distribution can be calculated analytically, enabling accurate numerical computation of the mutual information. Two cases were tested analytically. In both cases, the only source of noise is the downstream noise. The noise entropy *H*(*R*|*S*) is then equivalent for all *S* and the second term in Eq (23) is simply the entropy of the downstream noise distribution, $\frac{1}{2}\log_{2}(2\pi e\sigma_{\text{down}}^{2})$. The nonlinearity thus only affects the response entropy *H*(*R*). The first of the two nonlinearities considered was the cumulative distribution function of the stimulus distribution; the output of the nonlinearity is then a uniform distribution on [0, 1]. The response distribution is this uniform distribution convolved with the downstream noise distribution, giving

$$\begin{array}{c} p\left(r\right)=\frac{1}{2}\text{erf}\left(\frac{r}{\sqrt{2}\sigma_{\text{down}}}\right)-\frac{1}{2}\text{erf}\left(\frac{r-1}{\sqrt{2}\sigma_{\text{down}}}\right), \end{array}$$

where $\text{erf}\left(x\right)=2\pi^{-1/2}\int_{0}^{x}dt\;\exp\left(-t^{2}\right)$ is the error function. The second nonlinearity tested was a piecewise-linear nonlinearity, $f\left(s\right)=\Xi (\frac{s-z_{0}}{z_{1}-z_{0}})$. The response distribution can be computed exactly, but the expression is moderately lengthy, so it is omitted here. In both cases, numerical evaluation of *H*(*R*) can be done by direct numerical integration of *p*(*r*)log_2_ *p*(*r*).

Optimal nonlinearities obtained by maximizing the mutual information were in broad agreement with those found by minimizing the mean squared error, as observed in S2 Fig (compared to Fig 2) and S3 Fig (compared to Fig 5).

#### Signal-to-noise ratio

For a single channel, we define the signal-to-noise ratio (SNR) used in Fig 2 as

$$\begin{array}{c} \text{SNR}=\frac{var_{s}\;\left[E[r\;|s]\right]}{E_{s}\;\left[var[r\;|s]\right]}, \end{array}$$ (27)

where the innermost expectation (numerator) and variance (denominator) are taken over all responses *r* = *κm* + *ζ*, conditioned on the stimulus. The outer variance (numerator) and expectation (denominator) are then taken over the stimulus distribution. In terms of integrals over the rescaled nonlinearities defined above, the SNR may be written

$$\begin{array}{c} \text{SNR}=\frac{\left\langle \tilde{f}\left(y_{1}\right)\tilde{f}\left(y_{2}\right)\right\rangle -{\left\langle \tilde{f}\right\rangle }^{2}}{\left\langle {\tilde{f}}^{2}\right\rangle -\left\langle \tilde{f}\left(y_{1}\right)\tilde{f}\left(y_{2}\right)\right\rangle +\kappa \left\langle \tilde{f}\right\rangle +\sigma_{\text{down}}^{2}}. \end{array}$$ (28)

The covariance-like term $\langle \tilde{f}\left(y_{1}\right)\tilde{f}\left(y_{2}\right)\rangle$ has the same form as Eq (17), evaluated with $\rho_{\text{eff}}=\frac{\sigma_{s}^{2}}{\sigma_{s}^{2}+\sigma_{\text{up}}^{2}}$. It is not an actual covariance; it results from averaging $E_{\eta }{\left[f\left(s+\eta \right)|s\right]}^{2}$ over the stimulus, using the fact we can rewrite this term as $E_{\eta_{1}}\left[f\left(s+\eta_{1}\right)|s\right]E_{\eta_{2}}\left[f\left(s+\eta_{2}\right)|s\right]$, for two independent random variables *η*_1_ and *η*_2_.

### Parameter values used in figures

For all results, the stimulus is drawn from the standard normal distribution, and nonlinearity outputs are constrained to fall between 0 and 1.

For the simulations presented in this work, we swept over the parameters listed in Table 1. We chose a lower value of *σ*_down_ = 0.1 rather than 0 to limit the number of parameter sets for which all noise sources in the model were zero, as these sets frequently do not converge within a reasonable amount of time. Because our code is written in terms of the unrescaled nonlinearities, we swept over *ρ*_eff_ by fixing *σ*_s_ = 1 and *σ*_up_ = 2 and sweeping over the upstream noise correlation coefficient *ρ*_up_ = {− 0.25, 0.0625, 0.3750, 0.6875, 1.0}. We swept over a much finer range of *ρ*_up_ between 0.875 and 1 to resolve the splitting seen in Fig 6. For these cases, we only used one initial seed to speed up computation.

**Table 1:** Parameter values used in numerical solutions of the coupled integral equations determining the optimal nonlinearities *f*_1_(*z*) and *f*_2_(*z*).

*ρ*_eff_	0, 0.25, 0.5, 0.75, 1.0
*κ*	0, 0.25, 0.5, 0.75, 1.0
*σ*_down_	0.1, 0.25, 0.5, 0.75, 1.0
*ρ*_down_	-1.0, -0.75, -0.5, -0.25, 0, 0.25, 0.5, 0.75, 1.0

The upstream noise variance must be larger than the stimulus variance in order to achieve *ρ*_eff_ = 0. As the rescaled nonlinearities only depend on the *ρ*_eff_, *κ*, *σ*_down_ and *ρ*_down_, this choice only affects the absolute values of the MSE, which do depend on the ratio between *σ*_s_ and *σ*_up_. Despite the dependence of the MSE on *σ*_s_ and *σ*_up_, changing these parameters does not change which solutions are optimal for a fixed set of *ρ*_eff_, *κ*, *σ*_down_ and *ρ*_down_. This is because, for the optimal nonlinearities, the MSE works out to (in terms of the rescaled nonlinearities and decoding weights)

$$\begin{array}{c} \chi^{2}=\sigma_{s}^{2}\left[1-\frac{\sigma_{}^{2}}{\sigma_{s}^{2}+\sigma_{\text{up}}^{2}}\left({\tilde{D}}_{1}\langle y{\tilde{f}}_{1}\rangle +{\tilde{D}}_{2}\left\langle y{\tilde{f}}_{2}\right\rangle \right)\right]. \end{array}$$

The term ${\tilde{D}}_{1}\langle y{\tilde{f}}_{1}\rangle +{\tilde{D}}_{2}\langle y{\tilde{f}}_{2}\rangle$ is always positive because the decoding weights and averages always have the same sign. For fixed parameter values, it is only this term that varies between ON-OFF and ON-ON pairs. As the rescaled quantities only depend on $\rho_{\text{eff}},\;\kappa ,\;\sigma_{\text{down}}^{2},$ and *ρ*_down_ (for equal noise variances in each pathway), the exact values of $\sigma_{s}^{2}$ and $\sigma_{\text{up}}^{2}$ do not determine which class of solutions is optimal except through *ρ*_eff_. However, because the values of $\sigma_{s}^{2}$ and $\sigma_{\text{up}}^{2}$ do affect the overall MSE, the optimal solution may not perform significantly better than “nearby” sub-optimal nonlinearities; e.g., there may be a wide range of nonlinearities that give MSE within 1–5% of the optimal nonlinearity when the upstream noise variance is large. See Figs 2 and 5 in Results.

Similarly, the percentage difference in MSE between ON-OFF versus ON-ON strategies can vary depending on the size of *σ*_s_ and *σ*_up_. Fig 7 shows differences of up to 20% for *σ*_s_ = 1.0 and *σ*_up_ = 1.0 (we use a smaller value of *σ*_up_ here to show that the percent differences in MSE can be significiant; the default value of *σ*_up_ = 2.0 yields percent differences in MSE of up to about 5%).

For Fig 8 (comparison of methods for determining the optimal nonlinearity), the parameters used were: *σ*_up_ = 0.2, *κ* = 10^-3^, and *σ*_down_ = 0.2.

For Fig 2 (single pathway optimal nonlinearities) the parameters are listed in Table 2; parameters for Fig 3 (comparison of optimal and suboptimal nonlinearities) are listed in Table 3; parameters for Fig 5 (parallel pathway optimal nonlinearities) are listed in Table 4.

**Table 2:** Parameters used to generate data shown in Fig 2 (single pathway optimal nonlinearities).

		low noise SNR = 5	medium noise SNR = 1	high noise SNR = 0.1
**upstream noise dominant**	*σ*_up_	0.375	0.92	3.1
	*κ*	10^-3^	10^-3^	10^-3^
	*σ*_down_	0.05	0.05	0.05
**Poisson noise dominant**	*σ*_up_	0.05	0.05	0.05
	*κ*	0.0395	0.5	6.6
	*σ*_down_	0.05	0.05	0.05
**downstream noise dominant**	*σ*_up_	0.05	0.05	0.05
	*κ*	10^-3^	10^-3^	10^-3^
	*σ*_down_	0.135	0.4175	1.54

**Table 3:** Parameters used to generate data shown in Fig 3 (comparison of optimal and suboptimal nonlinearities).

**upstream noise dominant**	*σ*_up_	0.8
	*κ*	0
	*σ*_down_	0.005
**Poisson noise dominant**	*σ*_up_	0
	*κ*	0.2
	*σ*_down_	0.005
**downstream noise dominant**	*σ*_up_	0.05
	*κ*	0
	*σ*_down_	0.3

**Table 4:** Parameters used to generate data shown in Fig 5 (parallel pathway optimal nonlinearities).

		low noise	high noise
**upstream noise dominant**	*σ*_up_	0.85	0.85
	*ρ*_up_	0.9762	-0.9073
	*κ*	0.1	0.1
	*σ*_down_	0.1	0.1
	*ρ*_down_	0	0
**Poisson noise dominant**	*σ*_up_	0.25	0.25
	*ρ*_up_	-0.7	-0.7
	*κ*	0.25	0.9
	*σ*_down_	0.1	0.1
	*ρ*_down_	0	0
**downstream noise dominant**	*σ*_up_	0.25	0.25
	*ρ*_up_	-0.7	-0.7
	*κ*	0.1	0.1
	*σ*_down_	0.25	0.9
	*ρ*_down_	0	0

## Supporting Information

S1 Fig Optimal nonlinearities for different choice of noise at nonlinearity output stage. As the amount of noise associated with the nonlinear processing stage increases, optimal nonlinearities steepen regardless of detailed statistical structure of this noise. We compared Poisson, multiplicative Gaussian, and binomial noise sources. Poisson noise was parameterized as described in the manuscript. Multiplicative Gaussian noise was drawn from a Gaussian distribution with mean zero and variance equal to the nonlinearity output multiplied by a parameter controlling noise strength. This noise was then added to the output of the nonlinearity. Noise strengths in the Poisson and multiplicative Gaussian plots are directly comparable in the sense that the corresponding lines have identical variance at each response level. Binomial noise was generated by drawing from a binomial distribution, with the number of “events” (analogous to the number of available vesicles) determining the noise level (where a greater number of events results in lower noise) and probability of success of each event given by the output of the nonlinearity, ranging from 0 to 1. We then found optimal nonlinearities through simulations that maximize the mutual information (as described in Methods). For each of these types of noise, the optimal nonlinearity steepens as noise is increased (dark blue to light blue lines).
(EPS)

S2 Fig Optimal nonlinearities for a single pathway when one noise source dominates, found by maximizing mutual information (MI). Same as Fig 2, except that mutual information is maximized. Optimal nonlinearities found by maximizing MI are generally steeper than those found by minimizing the MSE of a linear estimator, but qualitative trends are the same. Nonlinearities are only shown for the two larger SNR values, as it was difficult to obtain reliable estimates of the mutual information for SNR = 0.1.
(EPS)

S3 Fig Optimal nonlinearities for a circuit with two parallel pathways, found by maximizing mutual information (MI). As in Fig 5, solid lines represent the optimal logistic nonlinearities (for opposite polarities in panel **A** and the same polarities in panel **B**), and shaded regions indicate the region that contains solutions within 1% of the maximum MI. For reference, dashed lines show analytic results obtained by minimizing the MSE of a linear estimator (identical to those in Fig 5). Optimal nonlinearities found by maximizing MI are steeper (as in S2 Fig), and additionally tend more towards independence in the two channels. However, qualitative trends are the same regardless of the specific criterion for optimization.
(EPS)
